# Carbon components in organic amendments drive nitrogen metabolism in one-year-long anaerobic soil microcosms

**DOI:** 10.3389/fmicb.2025.1588169

**Published:** 2025-06-02

**Authors:** Yiming Ma, Qiaoyu Wu, Xinhui Wang, Weikang Sui, Xiaojun Zhang

**Affiliations:** State Key Laboratory of Microbial Metabolism, Joint International Research Laboratory of Metabolic and Developmental Sciences, School of Life Sciences and Biotechnology, Shanghai Jiao Tong University, Shanghai, China

**Keywords:** metagenome, functional genes, microcosm experiment, nitrogen cycling, organic amendments

## Abstract

**Introduction:**

Long-term studies on the dynamic changes in nitrogen metabolism and functional microbial communities under anaerobic conditions, particularly those driven by organic amendments, remain scarce.

**Methods:**

We conducted a year-long anaerobic microcosm experiment using three organic amendments—aerobically fermented pig-manure digestate (ACM), compost (ACP) and straw powder (ACS)—alongside an inorganic fertilizer-only control (ACN).

**Results:**

Temporal shifts revealed that organic amendments drove distinct nitrogen metabolism pathways. Amendments of digestate and compost promoted the proliferation of nitrogen-mineralizing bacteria such as *Ramlibacter* and *Lysobacter*, leading to significant ammonium accumulation. After 12-month incubation, the ACM treatment caused a 75.6-fold increase in ammonium, a 43.4% rise in total nitrogen (TN), and a 27.0% increase in total organic carbon (TOC). In contrast, the ACS treatment exhibited superior nitrogen fixation, with an average of 1.69-fold higher rate than ACM and 5.30 fold higher than ACP The ACS treatment enriched cellulolytic nitrogen-fixing bacteria, including *Clostridium*, and nitrogen-fixing archaea.

**Discussion:**

This study provides profound insights in to the unique nitrogen metabolism pathways influenced by organic amendments under anoxic conditions, ultimately offering valuable insights into improved soil fertility and sustainable nitrogen management practices in agricultural systems.

## 1 Introduction

Nitrogen fertilizers have been widely applied in recent decades to support high agricultural crop yields (Ladha et al., 2016; Guo et al., 2022). However, excess fertilization and low utilization lead to several environmental problems, such as nitrous oxide (N_2_O) emissions (Li et al., 2024), ammonia volatilization (Ju and Zhang, 2017), and nitrate leaching (Zhao et al., 2022). Various management strategies to overcome the above issues have been evaluated (Young et al., 2021; Hassan et al., 2022; Gao and Cabrera Serrenho, 2023). Deeper application of fertilizers reduces ammonia volatilization by minimizing nitrogen exposure to the atmosphere (Ju and Zhang, 2017; Li et al., 2022), and simultaneously promote the formation of anaerobic micro-zones within the soil (Li et al., 2016; Schlüter et al., 2025). Additionally, combining organic amendments, such as manure (Wang et al., 2022), manure compost (Oyetunji et al., 2022), crop residue (Fu et al., 2021), and digestate (Samoraj et al., 2022; Chozhavendhan et al., 2023), with nitrogen fertilizers can improve soil fertility, enhance nitrogen use efficiency, and contribute to stable crop yields and environmental benefits (Selim, 2020; Liu et al., 2021a; Zhai et al., 2022; Liu et al., 2023).

Long-term field trials have revealed that organic amendment inputs contribute to soil organic carbon (SOC) stabilization by increasing the abundance of stable aliphatic and aromatic carbon compounds, as evidenced by solid-state nuclear magnetic resonance (solid-state NMR) spectroscopy (Guo et al., 2019; Yuan et al., 2025). Importantly, such carbon-fraction diversity is increasingly recognized as a key factor influencing soil microbial community composition, with potential consequences for biogeochemical cycling processes (Fierer, 2017). Material-driven heterogeneity, particularly variations in carbon fraction composition, has also been increasingly recognized as a critical driver of microbial community assembly and biogeochemical processes (Kallenbach et al., 2016; Liang et al., 2017). Changes in the soil carbon-to-nitrogen ratios (C/N) may regulate nitrogen immobilization and mineralization by influencing soil microbial community composition and the activities of microorganisms in decomposing organic matter fractions (Lin et al., 2019; Cui et al., 2022). In addition, exogenous organic matter can stimulate soil organic matter (SOM) mineralization through positive priming effects, enhancing nutrient availability in soil (Hicks et al., 2019). Several long-term field experiments have demonstrated that manure application promotes organic nitrogen accumulation and mineralization (Abubaker et al., 2012; Wu et al., 2017; Xia et al., 2021).

Deeper placement of fertilizers helps to create anaerobic zones, which consequently enhance denitrification and increase N₂O emissions (Meng et al., 2022). However, inconsistent findings regarding the impacts of combined inorganic and organic fertilization on N₂O emissions have been reported in literature (Reddy and Crohn, 2014; Akhtar et al., 2020; Zhang et al., 2023; Zuo et al., 2023; Wu et al., 2024). These discrepancies are likely due to variations in the types of organic amendments and differences in nitrogen-cycling microbial communities (He et al., 2019; Zhou et al., 2022). Thus, it is critical to investigate how organic amendments with distinct carbon compositions modulate the structure and function of nitrogen-transforming microbiomes under oxygen-limited conditions. Such insights are essential for unraveling the microbial mechanisms underlying nitrogen cycling and for informing fertilization strategies that improve nitrogen use efficiency while reducing environmental risks.

Previous studies have investigated the effects of organic amendments on soil microbial community compositions and nitrogen cycling functions (Liu et al., 2021b; Cui et al., 2022; Chen et al., 2023). However, most investigations have relied on short-term incubations or endpoint sampling, which provide limited insights into the long-term succession dynamics of nitrogen-transforming microbiomes (Ouyang et al., 2020; Bossolani et al., 2023; Pastorelli et al., 2024). While a few efforts have introduced a dynamic perspective, such as a 16-week anaerobic incubation tracking microbial community shifts under straw-amended soils (Yang et al., 2023). In that study, 16S rRNA gene-based taxonomic profiling was employed, but without any functional analysis.

Yet nitrogen transformation processes, such as denitrification, nitrogen fixation, and organic matter decomposition, typically involve slow and progressive shifts in microbial community composition and functional activity. Localized anaerobic conditions frequently arise in agricultural soils following deep fertilizer placement or intense microbial respiration, and the biogeochemical processes occurring within these zones unfold over extended periods. Consequently, short-term studies may fail to capture the cumulative functional succession of nitrogen-transforming microbiomes under oxygen-limited conditions, underscoring the need for long-term incubation experiments that better simulate these ecological dynamics. Nevertheless, the mechanisms by which amendment-derived carbon fraction differences drive the functional succession of nitrogen-transforming microbiomes under prolonged oxygen limitation remain poorly understood. To address these gaps, we designed a one-year-long anaerobic incubation experiment using *γ*-ray irradiated, carbon-normalized organic amendments with standardized nitrate additions, coupled with multi-time-point shotgun metagenomics and nitrogenous gas flux monitoring, aiming to elucidate the microbial mechanisms by which the discrepancy of amendment-derived carbon fractions drives nitrogen cycling under sustained oxygen limitation.

## 2 Materials and methods

### 2.1 Experimental materials

Soil samples were collected from the Shangzhuang Experimental Station (39°48′N, 116°28′E) of China Agricultural University, which was initiated in October 2006. The experimental site followed a winter wheat–summer maize rotation, with fertilizers applied based on basal and follow-up applications. The original soil used in this study was topsoil (0–20 cm) collected from the conventional fertilizer straw-no-return treatment group (Abbreviated C for original soil), passed through a 2 mm sieve, and stored at 4°C before subsequent experiments. The main properties of the soil were displayed in Supplementary Table S1.

Three types of organic amendments were used in this study: digestate remaining after anaerobic fermentation of swine manure for methane production (abbreviated as M), compost from aerobic fermentation of pig manure (abbreviated as P), and a 1:1 (w:w) mixture of wheat straw and corn straw powder (abbreviated as S). These amendments were selected for their relevance to agricultural practices and their distinct impacts on nitrogen cycling and microbial dynamics. Straw was chosen to simulate in-situ straw return, representing crop residues from the experimental field. Compost and digestate, both derived from pig manure, were selected due to their widespread use in agricultural practices. The differences in fermentation methods influence nitrogen release patterns and microbial community impacts.

The organic amendments were sterilized to exclude the introduction of exogenous bacteria before being added to the soil. Sterilization was conducted with cobalt 60 irradiation using a sterilization dose of 50 kGy by the Shanghai Radiant Technology Company, following established laboratory protocols and methods validated in previous studies (Wu et al., 2019). The main properties of the organic amendments were displayed in Supplementary Table S1.

### 2.2 Experimental design

To investigate the effects of adding organic matter with different carbon and nitrogen fractions on soil nitrogen metabolism, four experimental groups were established. The experimental groups consisted of anaerobically fermented pig manure digestate (ACM), aerobically fermented pig manure compost (ACP), straw powder (ACS), and a control group without organic matter amendment (ACN). In these group designations, ‘A’ represents anaerobic conditions, and ‘C’ refers to conventional nitrogen-fertilized soil.

Organic matters were added to the soil based on equivalent total carbon input to ensure that any observed effects were caused by differences in the composition of the amendments rather than differences of carbon content. The total amount of aerobically fermented pig manure compost, which had the highest nitrate content, was set to be added to 10% (w:w) of the soil (with an initial total carbon content of 8.992 g per kg of dry soil). The amounts of anaerobically fermented pig manure digestate and straw powder were calculated to match the total carbon content applied in the ACP treatment, ensuring consistency in carbon input across all treatments.

To control the unexpected effects of variations in carbon and nitrate content, we ensured consistency in the total carbon content and nitrate levels before the start of the incubation. This would ensure that observed differences in nitrogen metabolism and microbial community composition were solely due to variations in the carbon components of the amendments.

Organic amendment was introduced in two batches: the first at the start of the incubation and the second after six months of incubation. The total amount of organic matters addition was evenly divided between these two batches, maintaining equivalent carbon input across all treatments, as described above. This two-batch application was designed to simulate a basal and follow-up fertilization pattern commonly used in agricultural management. Potassium nitrate solution was added to maintain consistent nitrate levels across all treatments, accounting for the nitrate already present in the organic amendments.

The organic matters and potassium nitrate solution were added to 25 g of soil in serum bottles, and the headspace was replaced with pure helium (99.99%) to maintain anaerobic conditions. Anaerobic conditions were achieved by performing multiple rounds of gas replacement with helium, and the anaerobic level was confirmed through monitoring, which showed oxygen concentrations remained below detection limits throughout the experiment. Incubation was carried out at 25°C in the dark, with soil moisture maintained at 75% water holding capacity (WHC). Additional nitrate and water were injected every three months to sustain the experimental conditions. Periodic supplementation every 3 months mimicked the fertilization pulses typical in agricultural settings, allowing for a more realistic assessment of the impact of organic amendments on nitrogen metabolism.

The experiment followed a parallel replicated design with 18 replicates per group. Triplicate samples were destructively collected from each group at time point of 7 days (D7), 3 months (M3), 6 months (M6), 9 months (M9), and 1 year (M12), for high-throughput sequencing of the 16S rRNA gene (V3-V4 regions) and for physicochemical analysis, including total carbon (TC), total nitrogen (TN), total organic carbon (TOC), pH, nitrate, nitrite, and ammonium. Before each sampling, headspace gas in the system was measured using the ROBOT (Supplementary Figure S2), an instrument capable of continuously monitoring the gas concentrations in the headspace of the bottles (Molstad et al., 2007). Bacterial community composition and functional gene profiles were analyzed through metagenomic shotgun sequencing at 7 days, 6 months, and 1 year.

The detailed information of experimental design is provided in Supplementary Figures S1, S2 and Supplementary Table S2.

### 2.3 Physical and chemical analysis of soils and organic amendment

TC, TOC, and TN were analyzed with an elemental analyzer (Vario EL III/Isoprime). TOC was determined after removing inorganic carbon by hydrochloric acid fumigation (Ramnarine et al., 2011). Inorganic nitrogen contents were determined with the cadmium reduction column method for nitrate and nitrite (Huffman and Barbarick, 1981) and with the indophenol blue method for ammonium (Dorich and Nelson, 1983). Total organic nitrogen (TON) was calculated as the total nitrogen minus the inorganic nitrogen (nitrate, nitrite, and ammonium). pH was determined in a solution with a distilled water–soil ratio of 2.5:1 using a pH meter (Mettler Toledo, Switzerland).

The organic carbon fractions of the four experimental groups were determined at Day 0 following the addition of the corresponding organic matter using the 13C cross-polarization/total sideband suppression (CP/TOSS) method of solid-state NMR (Bruker Avance Neo 600 MHz). The NMR integrals of each functional group in the organic carbon fractions were calculated using the Topspin 4.13 program (Chen et al., 2018).

To compare the denitrifying potential of soil before and after 1-year incubation, the denitrification gas kinetics with addition of 150 mg N/kg of nitrate were assessed using the ROBOT system (Supplementary Figure S2) for multiple sampling during 3 day period (Molstad et al., 2007).

### 2.4 Calculation of nitrogen fixation rates

To quantify nitrogen fixation, we developed a calculation approach based on the difference between the total amount of added nitrate and the remaining nitrogen gas (N₂) concentration in each closed system. The calculation of nitrogen fixation was conducted separately for two stages, as the seal of the vials was opened in between. They are stages of the first 6 months (1) and the second 6 months (2), as indicated below.


(1) F1=A1–NG1

(2) F2=A2−NG2+F1

where (1) F1 represents the amount of nitrogen fixation of each group in the first stage, A1 represents the total amount of nitrate nitrogen added to each sample during the D0–M6 period (210 mg/kg), and NG1 represents the nitrogen content measured in each closed system at M6. (2) F2 represents the amount of nitrogen fixation of each group during the second stage, and A2 represents the total amount of nitrate nitrogen added to each sample during the M6–M12 period (120 mg/kg), while NG2 represents the nitrogen content measured in each closed system at M12.

### 2.5 16S rRNA gene amplicon sequencing and bioinformatics analysis

DNA was extracted from 0.5 g of soil samples as described previously (Griffiths et al., 2000; Paulin et al., 2013). Two rounds of polymerase chain reaction (PCR) were used to amplify the V3-V4 hypervariable regions of 16S rRNA genes from the soil DNA to construct high-throughput sequencing libraries (Zhang et al., 2016; Wu et al., 2019). The first round of PCR was performed using the universal primers B341F/B785R and the PCR products were purified by magnetic bead adsorption. The second round of PCR was performed using purified products as the template and primers with sequencing index tags added to the DNA amplicons obtained by the second round of PCR. The constructed sequencing libraries were then mixed in equal concentrations and subjected to high-throughput sequencing on the Illumina Miseq sequencing platform.

The obtained sequences were processed using the QIIME2 platform (Bolyen et al., 2019), and the “cutadapt” plug-in was used to excise junctions and primers, followed by the use of the DADA2 program to perform quality control of sequences (trimming, filtering, denoising, and chimera removal) and to merge amplicon sequence variants (ASVs) to obtain the original abundance matrix and ASV sequence information. A total of 1,949,178 high-quality sequences were obtained from 63 samples after quality control and filtration, and 9,561 ASVs were obtained after clustering (Supplementary Table S3). The sequence numbers of all samples were normalized to 17,000 (the number of sequences in the sample with the fewest sequences) to enable direct comparisons among samples. The subsequent analyses conducted in this study were based on normalized data. The ASV classifications were then taxonomically annotated using the SILVA138.2 database (Bolyen et al., 2019). The rarefaction curves (Supplementary Figure S7A) eventually flattened, indicating that the sequencing depth was sufficient to meet the requirements of the subsequent data analysis.

### 2.6 Metagenome shotgun sequencing and bioinformatics analysis

Soil metagenomic DNA was used for library construction and sequenced on an Illumina NovaSeq high-throughput sequencing platform (Personal Co. Ltd., Shanghai). A total of 39 samples were collected in triplicates from initial samples, and from four incubation groups in D7, M6 and M12 for metagenomic sequencing. Clean metagenomic sequencing reads were obtained after quality control using FastQC (Andrews, 2010), followed by assembly with the MEGAHIT (Li et al., 2015) to obtain contigs that were assembled into scaffolds using IDBA (Peng et al., 2010). Basic sequencing and quality control metrics for all samples, including raw data, GC content, Q30, and assembly statistics, are summarized in Supplementary Table S4. Gene prediction was then performed using the MetaGeneMark (Hyatt et al., 2012), followed by clustering of proteins using CD-HIT (Fu et al., 2012) into groups with >90% sequence similarity to enable removal of redundancy. The longest sequence was then used as the representative sequence to obtain non-redundant protein sets. The htseq-count was used to count the number of reads mapped to the non-redundant genes and assess gene abundances using transcripts per million (TPM) values. Nitrogen-related functional proteins were then annotated using the NcycDB based on the non-redundant protein datasets (Tu et al., 2019). Furthermore, carbohydrate degradation-related functional proteins (referred to as CAZymes) were annotated using the CAZy database (Drula et al., 2022), while taxonomic annotation was conducted using the NCBI non-redundant (NR) protein database.

Each sample was subjected to generate metagenome-assembled genome (MAG) using MetaBAT2 (Kang et al., 2019). The obtained MAGs were then quality-filtered using the CheckM (Parks et al., 2015). “High-quality draft genomes” with estimates of completeness >90% and contamination <5%, in addition to “medium-quality draft genomes” with estimates of completeness ≥50% and contamination <10%, were retained, for a total of 651 MAGs (Bowers et al., 2017). The genome datasets were subjected to de-redundancy using the dRep (Olm et al., 2017) based on 99% average nucleotide identity (ANI), leading to a total of 470 non-redundant MAGs. The relative abundances of non-redundant MAGs were calculated using CoverM (https://github.com/wwood/CoverM/releases/tag/v0.6.1). Taxonomic annotations of the non-redundant MAGs were then assigned using the GTDB-Tk (release 207) (Chaumeil et al., 2022). Phylophlan v.3.0 was subsequently used for phylogenetic tree construction (Asnicar et al., 2020), followed by visualization of trees using iTOL (Letunic and Bork, 2019). Nitrogen cycling gene annotations within MAGs were assigned using NcycDB, while annotations of lignin, cellulose, and hemicellulose degrading enzymes were assigned with the CAZy database. Kyoto Encyclopedia of Genes and Genomes (KEGG) Orthology (KO) annotation of MAGs for the KEGG database was conducted using kofam_scan v.1.3.0 (ver. 2023-03-02 KEGG release 105.0) (Aramaki et al., 2020). The KEGGDecoder was then used to parse the kofam_scan module outputs to determine the completeness of various metabolic pathways (Graham et al., 2018). Detailed sequencing and analysis parameters were provided in Supplementary Table S12.

### 2.7 Statistical analysis

Repeated measures Two-way analysis of variance (ANOVA) was applied to analyze the denitrification gas data (e.g., N₂O and N₂ dynamics) to assess the effects of organic amendment type and incubation duration across multiple time points. For other physicochemical indicators (e.g., ammonium concentration, nitrogen fixation rate), standard Two-way ANOVA was used. To meet ANOVA assumptions, logarithmic transformation was applied to the data where necessary, based on assessments of normality (Shapiro–Wilk test) and homogeneity of variances (Brown-Forsythe test). *Post hoc* comparisons were conducted using Tukey’s honestly significant difference (HSD) test to identify significant differences between group means (Ratsiatosika et al., 2024). All analyses were performed using GraphPad Prism 10 (Swift, 1997), with the significance level set at *α* = 0.05 for all tests. Full details of the statistical analysis are provided in Supplementary Tables S8–S11.

Principal coordinates analysis (PCoA) was calculated using R vegan package for Bray-Curtis distances and plotted using ggplot2 (Wickham, 2011) package. The overall statistical significance of group separation was assessed using permutational multivariate analysis of variance (PERMANOVA).

**Figure 1:**
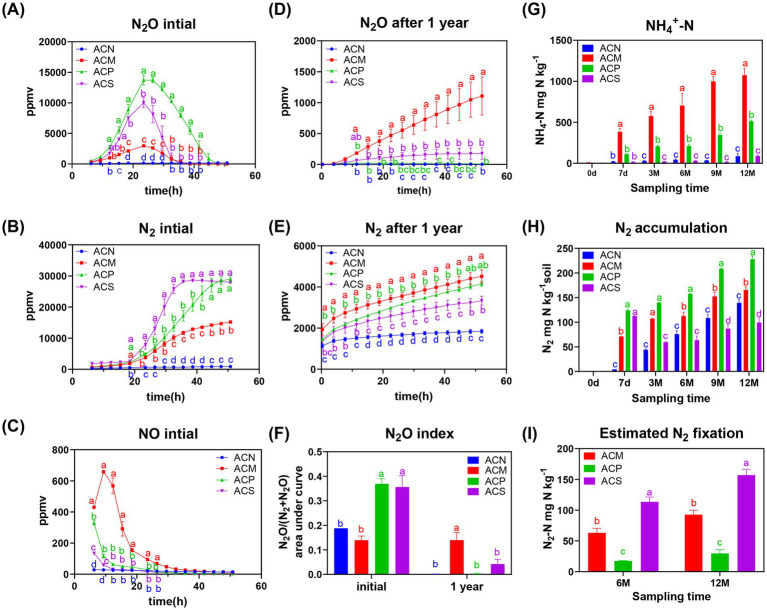
Dynamic variations in N_2_O, NH_4_^+^-N, N_2_, NO and nitrogen fixation activity during the one - year incubation. N_2_O **(A)**, N_2_ **(B)**, and NO **(C)** dynamics measured in soil sample before the1-year incubation. N_2_O **(D)** and N_2_ **(E)** dynamics measured in soil after the 1-year incubation. **(F)** N_2_O index before and after 1-year incubation. **(G)** Accumulation of ammonium during the incubation. **(H)** Accumulation of nitrogen gas during the incubation. **(I)** Amount of fixed nitrogen after 6 and 12 months of incubation. Error bars indicate the standard error of the means (*n* = 3). Significance was determined using repeated measures Two-way ANOVA for **(A–E)** and standard Two-way ANOVA for **(F–I)**, followed by Tukey’s HSD *post hoc* test. Different letters indicate significant differences between groups at each time period (*p* < 0.05). Global and inter-time point significance results are provided in Supplementary Tables S8, S10, and Supplementary Figure S6.

We constructed six random forest regression models using the R package ‘randomForest’ (Breiman, 2001) to identify MAGs associated with changes in ammonium content (Figure 1G), nitrogen fixation (Figure 1I), N₂O accumulation (Figures 1A,D), and total denitrification (N₂O + N₂, Figures 1B,E) across incubation stages. The significance of selected MAGs was assessed using the percentage increase in mean square error (MSE%), with higher values indicating greater importance (Jiao et al., 2018). Model performance was evaluated using the ‘A3’ package for cross-validation *R*^2^ and permutation significance tests, while predictor variable significance was assessed via ‘rfPermute’ (Jiao et al., 2018). Full model details and the importance and significance of selected MAGs are provided in Supplementary Tables S6, S7, respectively.

## 3 Results

### 3.1 Effects of different organic amendments on nitrogen metabolic processes

The three types of organic amendments differentially affected soil physicochemical parameters in Fluvo-aquic soils (Supplementary Figures S3–S5). Solid-state NMR measurement of composition of carbon fractions in the four experimental groups at day 0 of incubation indicated that all three organic matter additions increased the percentage of labile carbon fractions (Supplementary Figure S3B), as the TOC content of the ACM, ACP, and ACS groups were equally and significantly higher than that of ACN (*p* < 0.05, Supplementary Figure S4B). The N-alkyl functional group with a chemical shift of 45–65 ppm (Supplementary Figure S3C) was higher in the ACM than ACP.

The three organic amendments differentially affected nitrogen metabolism, specially, all of them enhanced soil denitrification capacity (Figures 1A–C). Furthermore, organic amendment significantly increased the production and accumulation of N_2_O and N_2_ at the beginning of incubation (*p* < 0.05, Figures 1A,B). After one year of incubation, N_2_ concentrations were high in all of the organic matter addition groups (Figure 1E), while only minimal N_2_O accumulation was detected in the ACM group (Figures 1D,F). At the beginning of the incubations, the ACP and ACS group treatments accumulated greater N_2_O, while the ACS group consumed nitrate more rapidly through denitrification (Figures 1A,B). Among the three organic treatments, ACM addition accumulated the least amount of N_2_O and consumed nitrate through denitrification most slowly (Figures 1A,B).

During the incubation period, ammonium significantly accumulated in the ACM and ACP groups with incubation time (*p* < 0.05, Figure 1G). Moreover, nitrogen balance calculations revealed that the amount of accumulated ammonium significantly exceeded the initial nitrate input (Supplementary Figure S5B), suggesting that the primary source of ammonium was the mineralization of organic nitrogen from the added organic amendments. In contrast, the ACS group exhibited an initial increase followed by a decrease in nitrogen levels, implying the presence of nitrogen fixation within the incubation systems (Figure 1H). The calculated nitrogen fixation suggested the highest levels in the ACS compared with the ACM and ACP groups, with a increasing trend observed within all treatments over time (Figure 1I).

### 3.2 Effects of different organic amendments on microbial communities

**Figure 2:**
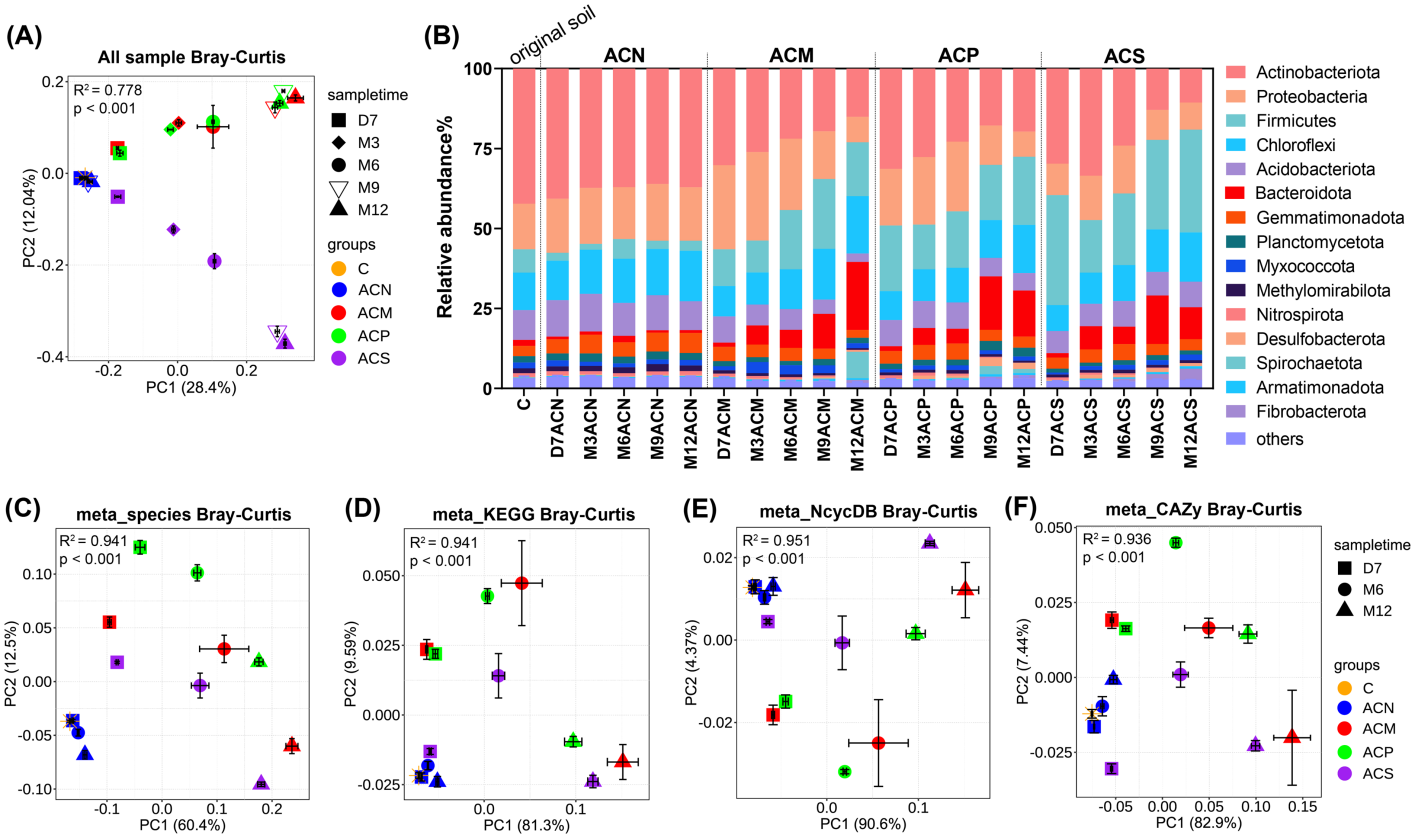
Microbial community compositional variation among treatments. **(A)** PCoA analysis of all samples based on Bray–Curtis distances of 16S rRNA gene sequence data. **(B)** Bacterial composition at the phylum level of samples. **(C)** PCoA analysis of microbial community composition based on species-level Bray–Curtis distances of metagenomic sequence data. **(D)** PCoA analysis of all KOs annotated based on kegg database. **(E)** PCoA analysis of functional genes related to the nitrogen cycle annotated based on NcycDB. **(F)** PCoA analysis of functional genes related to carbohydrate metabolism annotated based on CAZY database. All PCoA analyses were based on Bray–Curtis distances. The coordinates of each point in each PCoA plot were the mean of each group of samples, and the error bars indicate the standard error of the means (*n* = 3). Differences among groups were calculated via PERMANOVA **(A,C–F)**.

After one year of incubation, the Shannon diversity index was consistently lower in all experimental groups with organic amendments (ACM, ACP and ACS) than in the control group (ACN) (*p* < 0.05, Supplementary Figure S7B). Furthermore, PCoA analysis based on Bray–Curtis distances (Figure 2A) indicated that the type of organic amendment and incubation time significantly affected microbial community composition (*p* < 0.001, Supplementary Table S11). Consistently, differences in phylum-level compositions were observed among groups (Figure 2B and Supplementary Table S13). For example, the organic amendments notably increased Firmicutes abundance (*p* < 0.05, Supplementary Table S13). Microbial community compositions in different groups also showed observable changes with incubation time, with the exception of the ACN group in which the majority of phyla remained relatively stable. Overall, ACS communities exhibited different variation tendency compared to the communities of two manure-based groups, ACM and ACP.

### 3.3 Temporal effects of organic amendments on nitrogen metabolism genes

**Figure 3:**
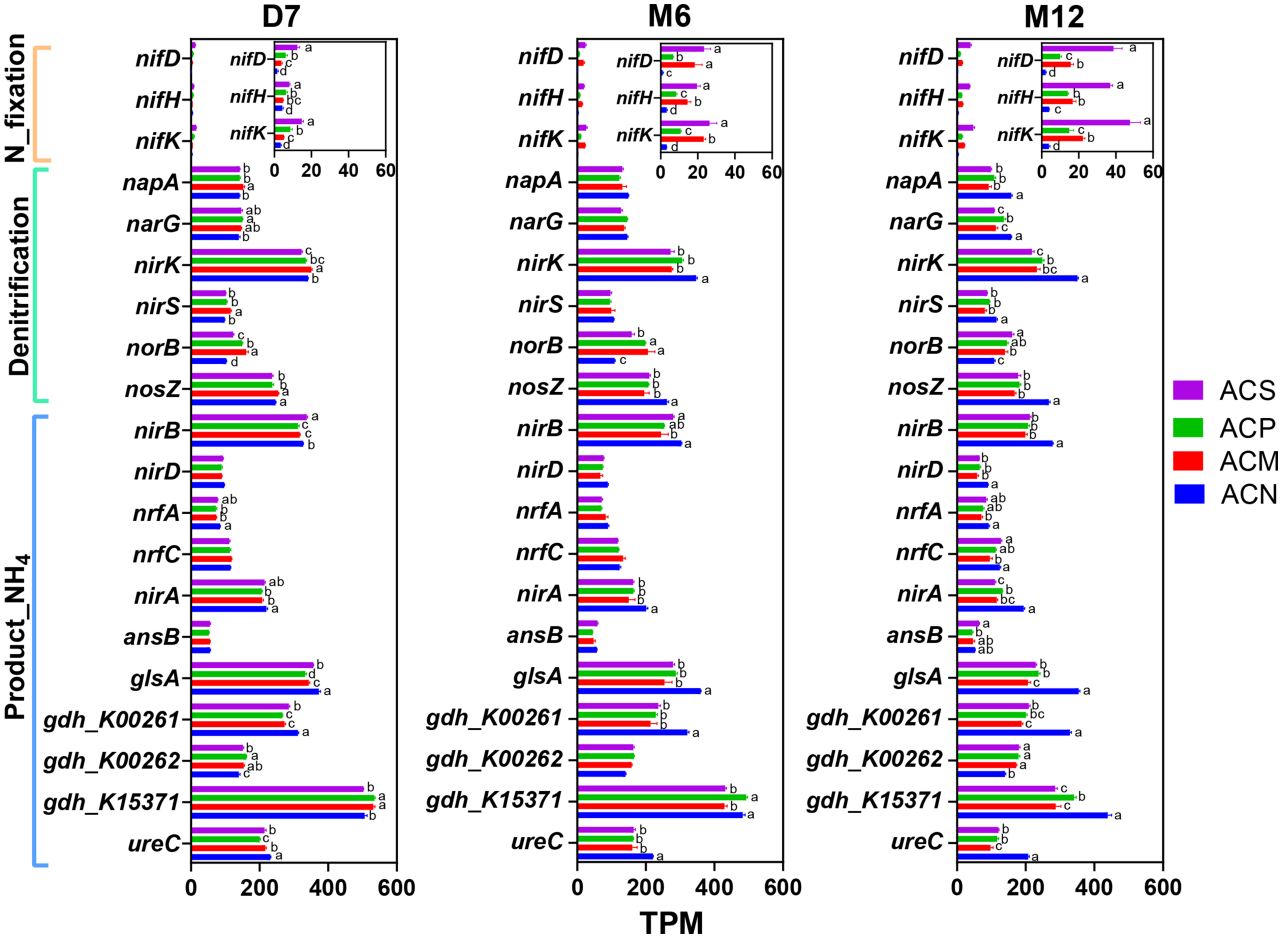
The abundances of functional genes for nitrogen fixation, denitrification and catalytic ammonium production function at different time points (D7: 7 days; M6: 6 months; M12: 12 months). Gene abundance in the metagenomic data was normalized and expressed as transcripts per million (TPM). Error bars indicate the standard error of the means (n = 3). Differences in the values were calculated based on analysis of variance (ANOVA) and Tukey’s HSD post hoc test. Different letters denote significant differences among groups at a significance level of *p* < 0.05.

From samples of this study, all sixty-eight nitrogen cycling genes annotated in NcycDB and associated with eight nitrogen cycling pathways were successfully identified in the metagenomic data (Supplementary Table S5). Among these pathways, Organic degradation and synthesis (ODAS), denitrification, Dissimilatory nitrate reduction (DNRA), and Assimilatory nitrate reduction (ANRA) showed consistently higher relative abundances across all samples (Supplementary Figure S8). Among these, two nitrogen mineralization functional genes (*gdh_K00262* and *gdh_K15371*) were identified in the ODAS pathway, which is associated with organic nitrogen degradation and ammonium production. Specifically, *gdh_K00262* was enriched over whole peroid in the experimental group with organic amendment, while *gdh_K15371* was enriched only in the early stage of incubation in ACM and ACP groups. In contrast, DNRA-related functional genes (*nirB*, *nirD*, *nrfA*, *nrfC*) and ANRA-related genes (*nirA*), which are associated with ammonium production, were consistently less abundant in the ACM and ACP groups compared to the ACN group (Figure 3). Furthermore, the total abundances of DNRA and ANRA pathway genes showed declining trends over time compared to the original soil (Supplementary Figures S8D,E). The nitrogen fixation genes *nifD*, *nifK*, and *nifH* were significantly enriched with incubation time in the ACS group (*p* < 0.05, Figure 3). In the organic amendment groups, enrichment of the denitrification functional genes *napA*, *nirK*, and *nosZ* decreased with incubation time (Supplementary Figure S9), while the relative abundances of *norB* genes significantly increased in these three groups (*p* < 0.05, Figure 3).

### 3.4 Effects of organic amendments on the functional microbiome of nitrogen mineralization and denitrification

**Figure 4:**
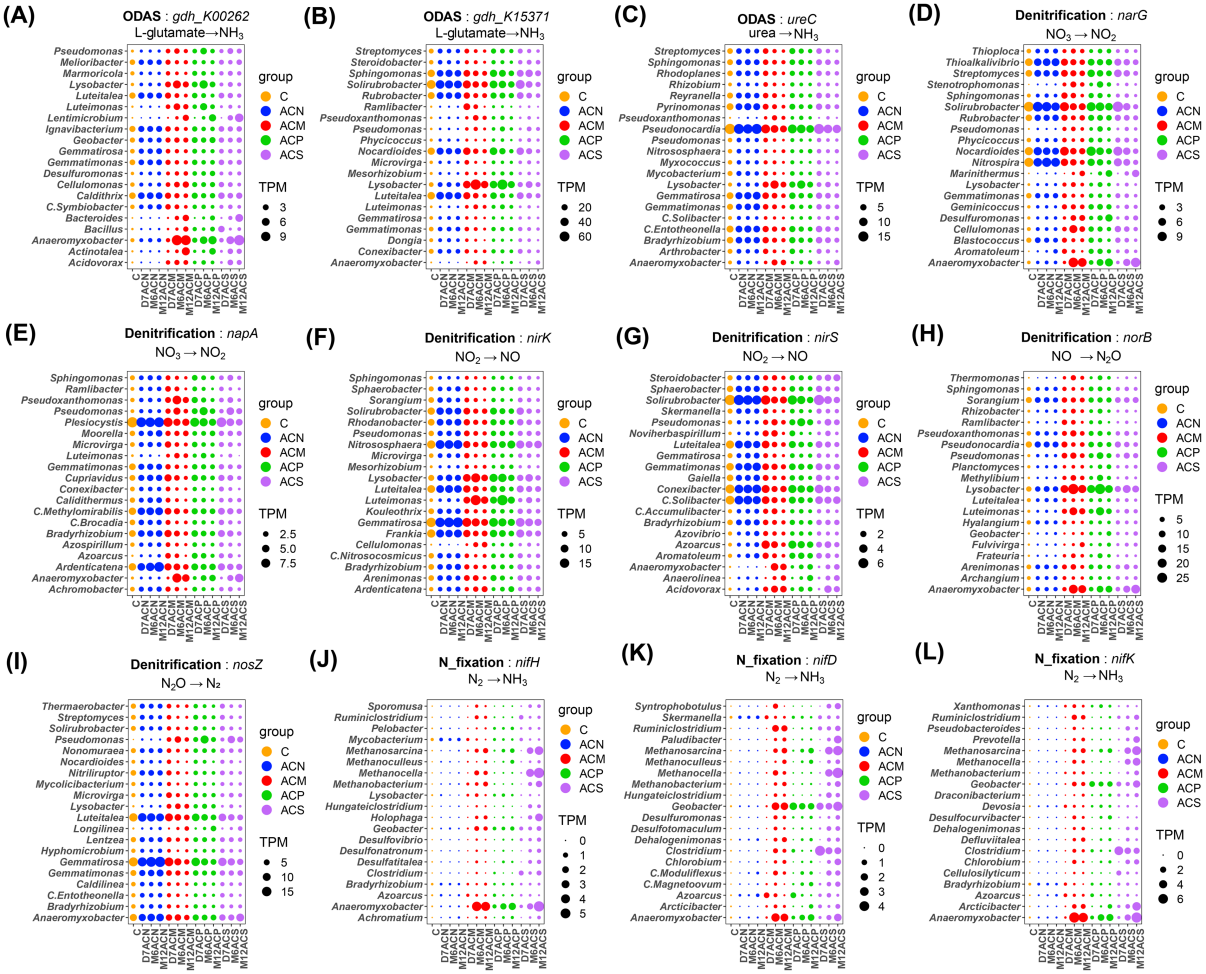
Top 20 dominant bacterial genera involved in nitrogen mineralization, denitrification and nitrogen fixation pathways. Dominant bacteria contain *gdh_K00262* **(A)**, *gdh_K15371* **(B)**, *ureC* **(C)**, *narG* **(D)**, *napA* **(E)**, *nirK* **(F)**, *nirS* **(G)**, *norB* **(H)**, *nosZ* **(I)**, *nifH* **(J)**, *nifD* **(K)**, *nifK* **(L)**, respectively. Circle size is the TPM value. Different colors represent different groups.

Considering the microorganisms that encoded nitrogen mineralization functional genes (Figures 4A–C), organic amendment enriched some specific taxa in all communities including *Lysobacter* and *Luteimonas*, in addition to the enrichment of *Anaeromyxobacter* and *Lentimicrobium* harboring the *gdh_K00262* genes at the later stage of cultivation. Organic matter addition also enriched *Anaeromyxobacter* that contains the denitrifying genes of *narG*, *napA*, and *norB* (Figures 4D,E,H). *Lysobacter* and *Luteimonas* harbor genes of *nirK*, *norB*, and *nosZ* (Figures 4F,H,I). In the microbial communities carrying the functional genes *gdh_K00262*, *gdh_K15371*, *ureC*, *nirK* and *nosZ*, compared with the ACN group, the abundances of *Luteitalea* and *Solirubrobacter* decreased across the incubation in the ACM, ACP, and ACS groups. Among the microorganisms carrying *nifH*, *nifD* and *nifK*, *Anaeromyxobacter* was enriched in organic amendment groups, while *Clostridium*, *Methanocella*, and *Methanosarcina* were only enriched in the ACS group (Figures 4J–L).

### 3.5 Diversity and nitrogen functional annotations of MAGs

**Figure 5:**
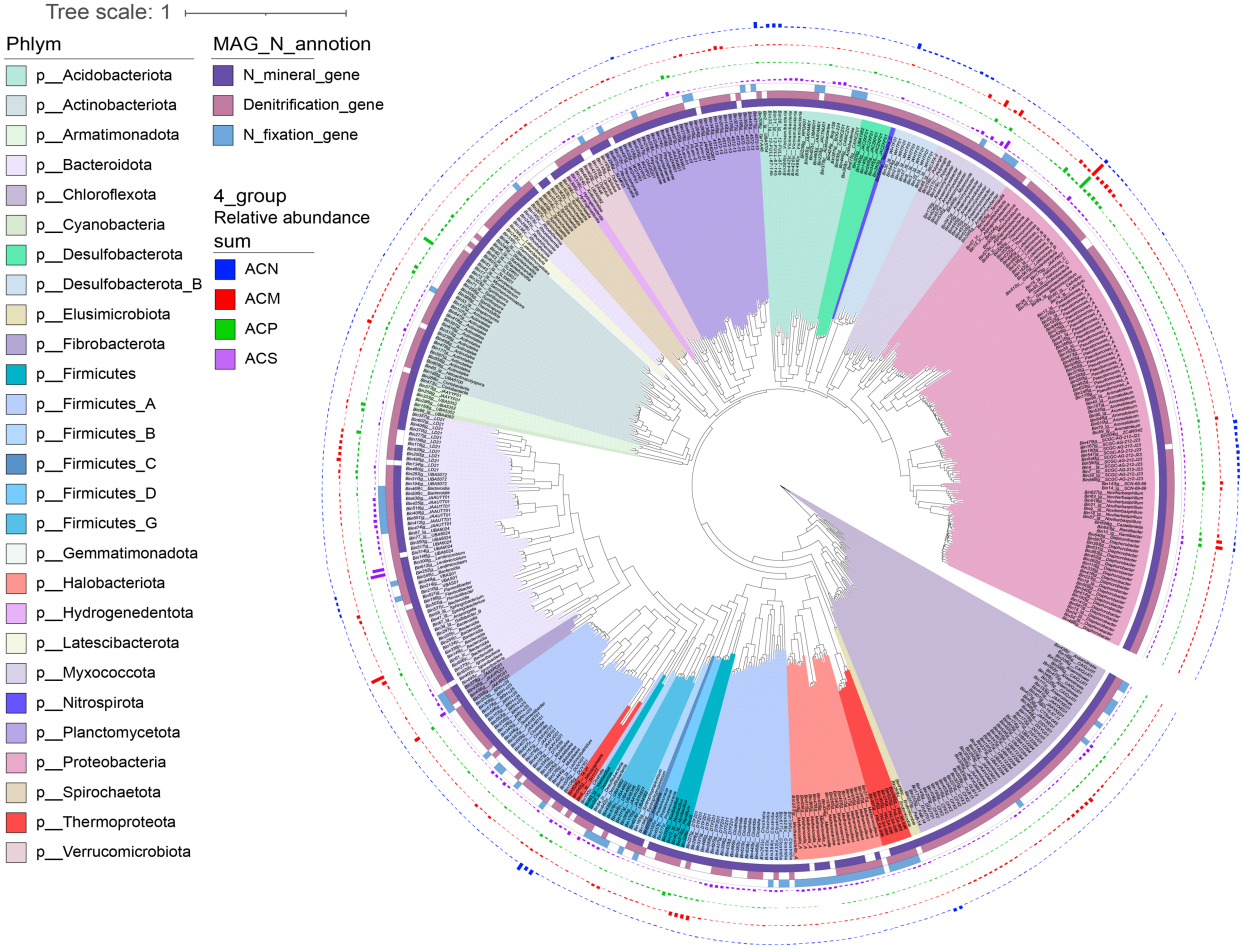
Phylogenetic tree showing the diversity, total abundances, and annotated nitrogen-related function of 470 reconstructed metagenome-assembled genomes (MAGs). Different colors on the phylogenetic tree branches represent different bacterial or archaeal phyla. The four outer circles of different colored bars represent the relative abundance of MAGs in the four experimental groups, with ACN in blue, ACM in red, ACP in green, and ACS in purple. The 3 inner circles indicate the types of nitrogen functional genes annotated in the MAG. The dark purple color indicates that a nitrogen mineralization gene is annotated, the light purple color indicates that a denitrification gene is annotated, and the light blue color indicates that a nitrogen fixation gene is annotated.

To better understand the links between microbial species and nitrogen cycling processes, we conducted metagenome binning to reconstruct MAGs. A total of 651 MAGs were reconstructed, of which 208 were high-quality draft genomes and 443 were medium-quality draft genomes (Bowers et al., 2017). 470 MAGs were retained after de-redundancy based on 99% ANI. The MAGs were assigned to 29 phyla, 47 classes, 84 orders (464 MAGs), 107 families (453 MAGs), and 105 genera (369 MAGs) based on annotation by the GTDB database (Figure 5). NcycDB-based functional annotation of nitrogen cycling genes indicated that 373 MAGs contained one or more functional genes for denitrification, while 443 MAGs contained genes related to ammonium production from organic nitrogen mineralization and 79 MAGs encoded functional genes for nitrogen fixation (Figure 5). Among all the 79 MAGs predicted for nitrogen fixation, 33 MAGs contained multiple cellulolytic and hemicellulolytic enzyme-encoding genes (Supplementary Figure S12), which were primarily derived from Bacteroidota, Fibrobacterota, and Firmicutes.

**Figure 6:**
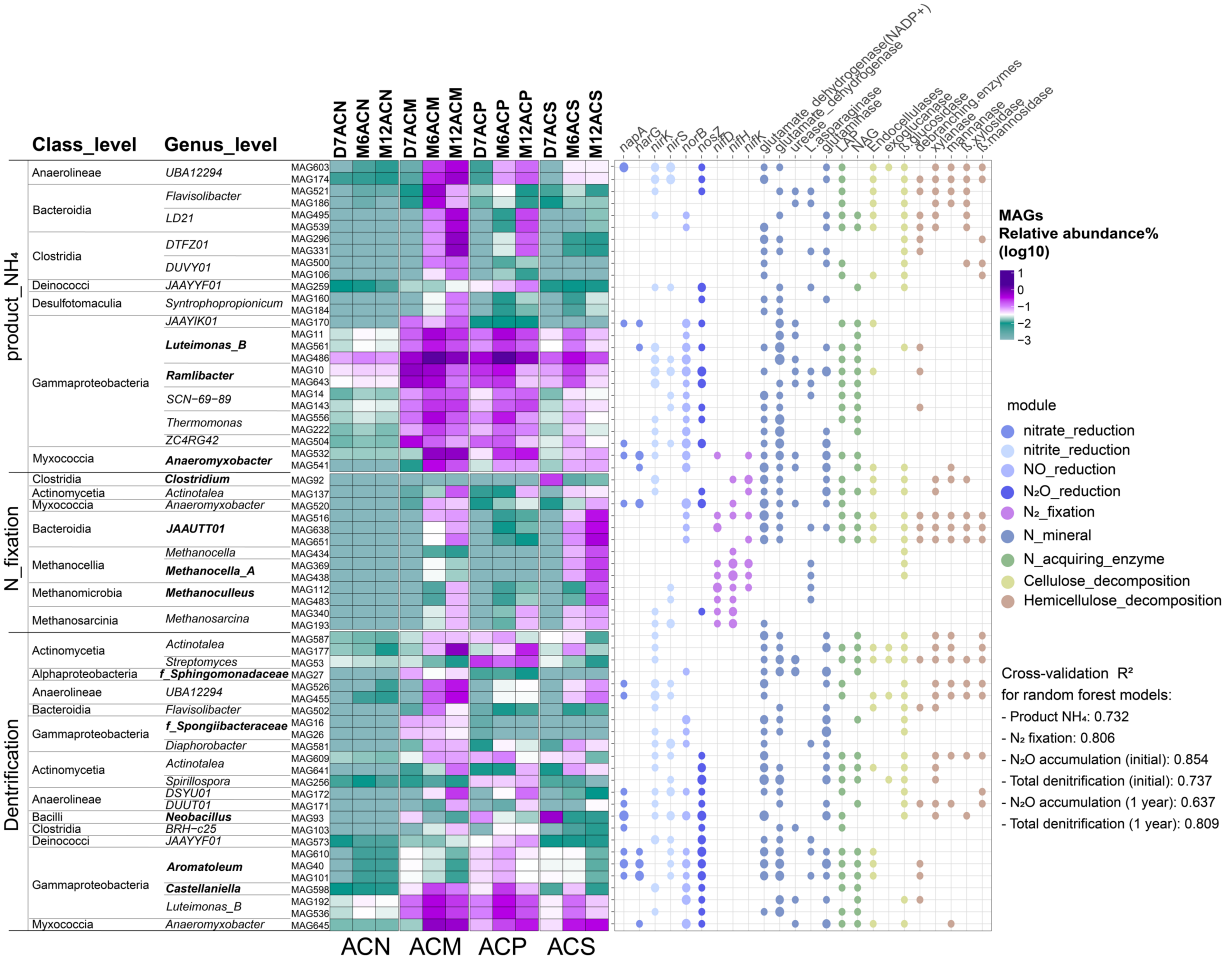
Random forest-selected metagenome-assembled genomes (MAGs) associated with the function of ammonium accumulation, nitrogen fixation, and denitrification. Heatmap indicates the relative abundance of the selected important MAGs. The bubble map represents the carbon and nitrogen-related functional genes annotated in the selected MAGs, different colors represent different modules. The cross-validation *R*^2^ values for the random forest regression models are as follows: ammonium accumulation (*R*^2^ = 0.73), nitrogen fixation (*R*^2^ = 0.81), and denitrification (*R*^2^ = 0.64 to 0.85 across different models). The details of the random forest regression model are displayed in Supplementary Table S6.

Twenty-six MAGs associated with changes in ammonium content were enriched in the ACM and ACP groups (Figure 6). In particular, MAGs primarily annotated as Burkholderiaceae, Xanthomonadaceae, and Anaeromyxobacteraceae were identified by the random forest regression model as important predictors contributing to ammonium accumulation. Among these, *Ramlibacter* exhibited higher relative abundance in the early cultivation stages, while *Luteimonas_B* had a higher relative abundance in the middle cultivation stage, and *Anaeromyxobacter* was more abundant in both the middle and late culture stages (*p* < 0.05, Supplementary Figure S13). Twelve MAGs that were important for changes in nitrogen fixation exhibited higher relative abundance in the late culture stage of the ACS group, with some also showing higher relative abundance at the late culture stage of the ACM group (Figure 6). Furthermore, MAGs with higher relative abundance at the late culture stage were primarily affiliated with the *JAAUTT01*, *Methanocella_A*, *Methanosarcina*, and *Methanoculleus* genera (*p* < 0.05, Supplementary Figure S13). Among the 25 MAGs associated with the denitrification process, 10 MAGs contained only N_2_O production genes and lacked *nosZ* for N_2_O reduction; these MAGs were primarily found in the ACM group (Figure 6). Additionally, *Neobacillus* MAG93 was identified as a complete denitrifier capable of converting nitrate to N_2_ and had high abundance in the ACS group at day 7 (*p* < 0.05, Supplementary Figure S13).

## 4 Discussion

### 4.1 Organic amendment and organic matter mineralization

During the incubation period, the ACM and ACP treatments resulted in significant ammonium accumulation, far exceeding the initial nitrate input (Supplementary Figure S5B). This results suggests that the primary source of ammonium derived from organic matter mineralization. The low abundances of DNRA and ANRA pathway-related genes (e.g., *nirB*, *nirD*, *nrfA*, *nirA*; Figure 3) and the overall declining trends of these pathways (Supplementary Figures S8D,E) further support their minimal contributions to ammonium accumulation. The increased gross nitrogen mineralization rates in these treatments were primarily attributed to one or more of several mechanisms, which are explored in the following sections.

First, the differences in the added organic matter fractions drove distinct nitrogen transformation pathways and fluxes. Solid-state NMR analysis indicated that the digestate and compost addition groups contained higher N-alkyl C fractions compared with the straw group, and this fraction primarily originated from proteins and amino acids (Chen et al., 2018). The rapid turnover of amino acids in soil (Jones and Kielland, 2012) may be one of the reasons for quick accumulation of mineralization-derived ammonium nitrogen in the ACM and ACP samples. By contrast, the higher percentage of the N-alkyl C component of ACM compared with the ACP group resulted in greater accumulation of ammonium in the ACM group. The stability index (Chen et al., 2018) relating to the ratio of alkyl-C to O-alkyl-C (A/O-A ratio) of the ACM group was lower than within the ACP group (Supplementary Figure S3D), indicating that its components were more easily degraded.

Second, manure additions greatly increased the abundance of the glutamate dehydrogenase encoding gene *gdh_k15371*, in addition to the abundances of the nitrogen-acquiring enzyme-encoding genes *K01255* (leucine aminopeptidase, LAP) and *K01207* (*β*-1,4-n-acetylglucosaminidase, NAG) during the early incubation stage (Supplementary Figure S10). The higher abundance of these functional genes during the initial stage suggests that the ACM and ACP groups had higher potential activities of organic nitrogen mineralizing enzymes and extracellular nitrogen-acquiring enzymes (Hu et al., 2020; Chen and Sinsabaugh, 2021). Nitrogen-acquiring enzyme activities have been shown to be positively correlated with nitrogen mineralization rates (Wang et al., 2023). These changes would increase the supply of dissolved organic nitrogen (DON) required for microbial nitrogen uptake and mineralization, as well as improve the ability to utilize organic nitrogen (Jian et al., 2016). Consistently with Wang et al. (2024), field applications of manure and digestate increased nitrogen-acquiring enzyme activities. This also aligns with the microbial nitrogen mining hypothesis (Craine et al., 2007), which suggests that excess exogenous carbon promotes microbial secretion of extracellular enzymes to acquire the nitrogen needed for growth.

Third, compost and digestate pig manure additions led to the enrichment of key bacterial taxa involved in nitrogen mineralization. MAGs associated with ammonium nitrogen production were enriched in the ACM and ACP groups. Among these, MAG10 and MAG643 of the genus *Ramlibacter*, which annotated contain the nitrogen-acquiring enzyme LAP encoding gene *K01255*, were most abundant in the early stage of incubation when ammonium accumulation rate was at its peak. Random forest regression analysis identified these MAGs as important predictors of ammonium levels. The abundance of genus *Ramlibacter* was also significantly and positively correlated with ammonium accumulation in another study (Hu et al., 2023).

### 4.2 Nitrogen fixation triggered by straw addition

The significant amount of nitrogen fixed in the ACS group could be due to the combined involvement of cellulolytic nitrogen-fixing bacteria (CNFB) and methanogenic archaea.

Straw addition enriched CNFB (Harindintwali et al., 2020), which produce cellulases and nitrogen-fixing enzymes, facilitating the degradation of cellulose into simple sugars and promoting nitrogen fixation. CNFB have been widely studied in agricultural composting due to their cellulose-degrading and nitrogen-enriching functions (Harindintwali et al., 2020; Harindintwali et al., 2022). Lignocellulose is a carbon-rich component of crop residues such as straw that primarily comprises cellulose, hemicellulose, and lignin (Harindintwali et al., 2020). Bacteria encoding *nifH*, *nifD*, and *nifK* were enriched in the ACS group, as represented by the anaerobic cellulose-degrading *Clostridium* (Figures 4J–L), with species having cellulolytic and nitrogen-fixing functions (McDonald et al., 2012; Poehlein et al., 2017). These bacteria may play a key role in both straw degradation and nitrogen fixation in the ACS group during early incubation. The D7ACS group was enriched in CAZymes involved in cellulose and hemicellulose catabolism (Bredon et al., 2018) (Supplementary Figure S11). Consequently, straw addition was hypothesized to rapidly promote the abundance of bacteria encoding cellulose and hemicellulose catabolic enzyme, such as MAG92 (Figure 6).

Nitrogen-fixing taxa enriched in the ACS group in mid-to late-culture periods comprised several methanogenic archaea, including *Methanocella* and *Methanosarcina*. The end products of the anaerobic degradation of lignocellulose are primarily carbon dioxide and methane (Nguyen and Khanal, 2018; Song et al., 2023). Methanogenic archaea in confined anaerobic systems use the products from the final step of lignocellulose fermentation, such as acetic acid, hydrogen, and carbon dioxide, to produce methane (Evans et al., 2019). These organisms were enriched over time in this study. Nitrogen fixation abilities had been documented in some methanogenic archaea (Belay et al., 1984; Murray and Zinder, 1984; Bomar et al., 1985; Scherer, 1988), although it is not a common trait among all methanogenic archaea. It is consistent with the result that the methanogen MAGs from the ACS group encoded genes for nitrogen fixation. The enrichment of these organisms should promote nitrogen fixation and methanogenesis by using fermentation products such as short fatty acids.

However, although this study provides insights into the regulation of soil N-functional communities by exogenous organic matter additions, we recognize that there are some limitations in the study. Our study did not adequately consider all factors that may affect the soil microbial community and N cycling. The factors, such as aeration in soil and plant cultivation and so on, deserve to be further explored in future studies for a more comprehensive understanding of the complexity of soil ecosystems. Despite these limitations, our findings offer promising insights for practical applications. The rapid accumulation of ammonium from anaerobically fermented pig manure digestate highlights its potential as a readily available nitrogen source to meet the early-stage nutrient demands of plants in agricultural cropping systems. Conversely, the enrichment of nitrogen-fixing CNFB and methanogenic archaea in straw-amended soils promoted long-term nitrogen fixation under anaerobic conditions. These results highlight the opportunity to optimize organic amendment strategies, balancing immediate nutrient availability with sustained nitrogen-fixing capacity in soils.

## 5 Conclusion

Adding compost, digestate, or straw to soil differentially shapes nitrogen (N) cycling via microbial mechanisms. Compost/digestate boosted microbial enzymes for nitrogen acquisition and ammonium accumulation via mineralization, while straw enriched methanogenic nitrogen-fixing archaea. These organic inputs create distinct nitrogen metabolism pathways by altering microbial communities and functional genes, ultimately affecting soil N availability and greenhouse gases. Strategically selecting organic amendments could synchronize nutrient release with crop demands while suppressing nitrous oxide emissions. This microbial-driven nitrogen partitioning offers a framework to optimize organic input timing/location, balancing soil fertility and environmental sustainability in intensive agriculture. Digestate provides a readily available nitrogen source for early-stage plant growth, while straw promotes long-term nitrogen fixation under anaerobic conditions. By selecting appropriate organic amendments based on the actual soil conditions, farmers may benefit from optimizing nitrogen cycling and improve soil sustainability.

## Data Availability

The data presented in the study are deposited in the NCBI Sequence Read Archive repository, accession number PRJNA1013701.
