# Supplementary material for: Carbon components in organic amendments drive nitrogen metabolism in one-year-long anaerobic soil microcosms
Source: Front Microbiol. 2025 Jun 2;16:1588169. doi: 10.3389/fmicb.2025.1588169 (PMC12177738; doi:10.3389/fmicb.2025.1588169)
Supplement: Supplementary file 1 [file Table_1.docx]

Supplementary Material

# Supplementary Figures and Tables

Files in Supplementary Information: Supplementary Table. S1-Table. S12

Supplementary Fig. S1-Fig. S13

## Supplementary Tables

**Supplementary Table S1** Physicochemical parameters of soil and organic amendments used in this experiment.

|  | Soil | Organic Matter | | |
| --- | --- | --- | --- | --- |
|  | Conventional fertilizer soil (C) | Anaerobically fermented pig manure digestate (M) | Aerobically fermented pig manure compost (P) | Straw powder (S) |
| Soil type | calcareous  Fluvo-aquic soil | - | - | - |
| Soil bulk density | 1.31 g cm^–3^ |  |  |  |
| Soil texture | clay loam  (28% clay, 32% silt and 40% sand  (USDA standard)^f^) | - | - | - |
| Latitude and longitude | 39°48′N, 116°28′E | - | - | - |
| Cropping system | Winter wheat, Summer maize | - | - | - |
| N Fertilizer input (kg N ha^−1^yr^-1^) | 560 (300 in Winter wheat 260 in Summer maize) | - | - | - |
| Water content (%) | 15.36±0.098 | 110.96±0.169 | 29.52±0.037 | 0 |
| WHC^a^ (%) | 35.34±0.263 | - | - | - |
| TC^b^ (% of dry weight) | 1.115±0.035 | 19.79±0.015 | 17.90±0.795 | 42.42±0.265 |
| TOC^c^ (% of dry weight) | 0.831±0.022 | 19.79±0.015 | 17.90±0.795 | 42.42±0.265 |
| TN^d^ (% of dry weight) | 0.08 | 1.64±0.02 | 1.87±0.05 | 0.79±0.015 |
| NO_3_^-^ -N (mg/kg) | 27.83±0.519 | 11.88±4.971 | 1128.81±37.492 | 225.80±2.175 |
| NH_4_^+^-N (mg/kg) | 6.95±0.921 | 2.61±0.826 | 0.81±0.109 | - |
| pH | 7.76±0.067 | 8.06±0.007 | 7.80±0.017 | - |
| C:N^e^ | 10.39±0.27 | 9.565±0.17 | 12.066±0.156 | 54.057±1.371 |

Values represent the mean ± standard deviation (*n* = 3)

**a.** WHC: Water Holding Capacity;

**b.** TC: Total Carbon;

**c.** TOC: Total Organic Carbon;

**d.** TN: Total Nitrogen;

**e.** C:N: Carbon-to-Nitrogen ratio (the ratio of TOC to TN);

**f.** USDA standard: United States Department of Agriculture Standard

Soil bulk density, texture data were retrieved from (Song et al., 2019), N Fertilizer input data were retrieved from (Huang et al., 2017)

**Supplementary Table S2** Nitrate and organic amendments added to each group at the corresponding sampling time point.

| **Fertilization**  **time** | **Goup** | **Soil weight (dry)**  **(g)** | **Weight of organic amendment added (dry)**  **(g)** | **Total NO_3_^-^-N added**  **(including**  **organic amendments)** |
| --- | --- | --- | --- | --- |
| 0 day | ACN | 25 | 0 | 150 mg N Kg^-1^ |
|  | ACP |  | 1.25 |  |
|  | ACM |  | 1.15 |  |
|  | ACS |  | 0.527 |  |
| 3 Month ^a^ | ACN | 25 | - | 60 mg N Kg^-1^ |
|  | ACP |  | - |  |
|  | ACM |  | - |  |
|  | ACS |  | - |  |
| 6 Month ^a,b^ | ACN | 25 | 0 | 75 mg N Kg^-1^ |
|  | ACP |  | 1.25 |  |
|  | ACM |  | 1.15 |  |
|  | ACS |  | 0.527 |  |
| 9 Month ^a^ | ACN | 25 | - | 60 mg N Kg^-1^ |
|  | ACP |  | - |  |
|  | ACM |  | - |  |
|  | ACS |  | - |  |

a: Fertilizers were applied at each time point to parallel culture samples remaining after destructive sampling

b: Open the culture system at 6 months when the organic improver is added again. Add organic amendment, mix well and reseal, replace headspace gas with helium.

**Supplementary Table S3** Overall quality of high-throughput sequencing data of 16S rRNA v3-v4 region of all samples based on QIIME2 analysis.

| **Table summary** | |
| --- | --- |
| Metric | Sample |
| Number of samples | 63 |
| Number of features | 9,561 |
| Total frequency | 1,949,178 |
| **Frequency per sample** | |
|  | Frequency |
| Minimum frequency | **17,338.00** |
| 1st quartile | 28,142.00 |
| Median frequency | 30,899.00 |
| 3rd quartile | 34,397.00 |
| Maximum frequency | 41,230.00 |
| Mean frequency | 30,939.33 |

A total of 1,949,178 high-quality sequences were obtained from 63 samples after quality control and filtration, and 9,561 ASVs were obtained after clustering. To accurately assess the diversity of the microbial communities, the number of sequences in different samples was normalized to 17,000 (the number of sequences in the sample with the fewest sequences). The subsequent analyses conducted in this study were based on normalized data.

**Supplementary Table S4** Metagenomic sequencing data quality control information.

| **sample_ID** | **raw data**  **(bp)** | **HQData**  **(bp)** | **HQData**  **(%)** | **GC**  **(%)** | **Q30**  **(%)** | **Contigs**  **Total**  **（bp）** | **Contigs**  **N50 length** |
| --- | --- | --- | --- | --- | --- | --- | --- |
| C1 | 20729705100 | 20610435366 | 99.42 | 65.04 | 95.1 | 1425043500 | 480 |
| C2 | 21876463800 | 21761670107 | 99.48 | 65.14 | 95.31 | 1580464912 | 478 |
| C3 | 20040925200 | 19919702367 | 99.4 | 64.61 | 94.84 | 1344582750 | 472 |
| D7ACM1 | 22082338500 | 21962639399 | 99.46 | 64.71 | 95.29 | 1614869186 | 491 |
| D7ACM2 | 20650616700 | 20545715960 | 99.49 | 64.97 | 95.33 | 1606744500 | 488 |
| D7ACM3 | 24588455100 | 24442050215 | 99.4 | 64.98 | 95.03 | 1832046048 | 514 |
| D7ACN1 | 19402752300 | 19306554556 | 99.5 | 64.67 | 95.11 | 1352932434 | 474 |
| D7ACN2 | 22769490600 | 22629182712 | 99.38 | 64.97 | 94.7 | 1547402516 | 485 |
| D7ACN3 | 20338954200 | 20196527779 | 99.3 | 64.89 | 94.46 | 1356901776 | 475 |
| D7ACP1 | 20246196300 | 20131412025 | 99.43 | 64.15 | 95.23 | 1419458802 | 501 |
| D7ACP2 | 22579649700 | 22455950956 | 99.45 | 63.47 | 95.05 | 1569325648 | 522 |
| D7ACP3 | 20484299400 | 20383694764 | 99.51 | 63.91 | 95.33 | 1461822188 | 502 |
| D7ACS1 | 18218860200 | 18146516979 | 99.6 | 62.39 | 95.73 | 1266030837 | 483 |
| D7ACS2 | 16735281300 | 16665526895 | 99.58 | 61.99 | 95.38 | 1063859873 | 484 |
| D7ACS3 | 16221598500 | 16149613020 | 99.56 | 62.42 | 95.21 | 1121096299 | 483 |
| M6ACM1 | 16128352500 | 16058594924 | 99.57 | 61.6 | 95.32 | 1217450960 | 587 |
| M6ACM2 | 18780046800 | 18701508908 | 99.58 | 62.89 | 95.51 | 1356946194 | 507 |
| M6ACM3 | 19358259300 | 19267841541 | 99.53 | 61.56 | 95.06 | 1426097126 | 614 |
| M6ACN1 | 17263052400 | 17191756070 | 99.59 | 64.71 | 95.46 | 1166704188 | 481 |
| M6ACN2 | 19651785600 | 19565858047 | 99.56 | 64.23 | 95.32 | 1413890347 | 479 |
| M6ACN3 | 17312794200 | 17232848930 | 99.54 | 64.45 | 95.15 | 1273817974 | 477 |
| M6ACP1 | 20500894500 | 20420877132 | 99.61 | 63.27 | 95.56 | 1624733343 | 542 |
| M6ACP2 | 22275964500 | 22194910318 | 99.64 | 63.52 | 95.69 | 1435840709 | 524 |
| M6ACP3 | 19409976900 | 19339160480 | 99.64 | 63.11 | 95.43 | 1254302854 | 538 |
| M6ACS1 | 18674568300 | 18613594816 | 99.67 | 60.76 | 96.03 | 1273150240 | 533 |
| M6ACS2 | 17199504900 | 17143769545 | 99.68 | 61.29 | 95.8 | 1173246677 | 561 |
| M6ACS3 | 19002501600 | 18938004618 | 99.66 | 61.98 | 95.93 | 1281754234 | 521 |
| M12ACM1 | 15971101200 | 15935300857 | 99.78 | 56.55 | 96.38 | 936487636 | 655 |
| M12ACM2 | 19973287500 | 19890227171 | 99.58 | 58.64 | 96.11 | 1496902246 | 670 |
| M12ACM3 | 20954423100 | 20856918992 | 99.53 | 59.94 | 95.87 | 1779948117 | 656 |
| M12ACN1 | 18657460800 | 18589434752 | 99.64 | 64.36 | 96.11 | 1291490008 | 481 |
| M12ACN2 | 21845985000 | 21766861619 | 99.64 | 64.46 | 96.07 | 1474036694 | 490 |
| M12ACN3 | 19698839100 | 19634278780 | 99.67 | 63.82 | 96.3 | 1423725838 | 485 |
| M12ACP1 | 20926595100 | 20846018558 | 99.61 | 59.72 | 96.11 | 1919072336 | 630 |
| M12ACP2 | 22217855400 | 22117202383 | 99.55 | 59.94 | 95.87 | 2032632699 | 640 |
| M12ACP3 | 20151816600 | 20057526314 | 99.53 | 58.98 | 95.7 | 1685595413 | 627 |
| M12ACS1 | 18902541600 | 18825859001 | 99.59 | 57.84 | 96.15 | 1603849209 | 671 |
| M12ACS2 | 17137455600 | 17071430131 | 99.61 | 58.17 | 96.11 | 1531958257 | 645 |
| M12ACS3 | 18186200700 | 18106834525 | 99.56 | 58.08 | 95.88 | 1566516657 | 707 |

**Supplementary Table S5** Information of all nitrogen cycle functional genes contained in NcycDB.

| **N cycling Pathway** | **NcycDB_Gene (sub) families** | **NcycDB_Annotation** | **KEGG_KO** | **KEGG_Symbol** |
| --- | --- | --- | --- | --- |
| Dissimilatory nitrate reduction (DNRA) | *napA^*^* | Periplasmic nitrate reductase NapA | K02567 | *napA* |
|  | *napB^*^* | Cytochrome c-type protein NapB | K02568 | *napB* |
|  | *napC^*^* | Cytochrome c-type protein NapC | K02569 | *napC* |
|  | *narG^*^* | Nitrate reductase | K00370 | *narG, narZ, nxrA* |
|  | *narH^*^* | Nitrate reductase | K00371 | *narH, narY, nxrB* |
|  | *narI^*^* | Nitrate reductase gamma subunit | K00374 | *narI, narV* |
|  | *narV^*^* | Nitrate reductase 2, gamma subunit | K00374 | *narI, narV* |
|  | *narJ^*^* | Nitrate reductase molybdenum cofactor assembly chaperone | K00373 | *narJ, narW* |
|  | *narW^*^* | Nitrate reductase 2, delta subunit | K00373 | *narJ, narW* |
|  | *narY^*^* | Nitrate reductase 2, beta subunit | K00371 | *narH, narY, nxrB* |
|  | *narZ^*^* | Nitrate reductase 2, alpha subunit | K00370 | *narG, narZ, nxrA* |
|  | *nirB* | Nitrite reductase (NADH) large subunit | K00362 | *nirB* |
|  | *nirD* | Nitrite reductase (NADH) small subunit | K00363 | *nirD* |
|  | *nrfA* | Nitrite reductase (cytochrome c-552) | K03385 | *nrfA* |
|  | *nrfB* | Cytochrome c-type protein NrfB | K04013 | *nrfB* |
|  | *nrfC* | Protein NrfC | K04014 | *nrfC* |
|  | *nrfD* | Protein NrfD | K04015 | *nrfD* |
| Denitrification | *napA^*^* | Periplasmic nitrate reductase NapA | K02567 | *napA* |
|  | *napB^*^* | Cytochrome c-type protein NapB | K02568 | *napB* |
|  | *napC^*^* | Cytochrome c-type protein NapC | K02569 | *napC* |
|  | *narG^*^* | Nitrate reductase | K00370 | *narG, narZ, nxrA* |
|  | *narH^*^* | Nitrate reductase | K00371 | *narH, narY, nxrB* |
|  | *narI^*^* | Nitrate reductase gamma subunit | K00374 | *narI, narV* |
|  | *narV^*^* | Nitrate reductase 2, gamma subunit | K00374 | *narI, narV* |
|  | *narJ^*^* | Nitrate reductase molybdenum cofactor assembly chaperone | K00373 | *narJ, narW* |
|  | *narW^*^* | Nitrate reductase 2, delta subunit | K00373 | *narJ, narW* |
|  | *narY^*^* | Nitrate reductase 2, beta subunit | K00371 | *narH, narY, nxrB* |
|  | *narZ^*^* | Nitrate reductase 2, alpha subunit | K00370 | *narG, narZ, nxrA* |
|  | *nirK* | Nitrite reductase (NO-forming) | K00368 | *nirK* |
|  | *nirS* | Nitrite reductase (NO-forming) | K15864 | *nirS* |
|  | *norB* | Nitric oxide reductase subunit B | K04561 | *norB* |
|  | *norC* | Nitric oxide reductase subunit C | K02305 | *norC* |
|  | *nosZ* | Nitrous-oxide reductase | K00376 | *nosZ* |
| Assimilatory nitrate reduction (ANRA) | *narB* | Assimilatory nitrate reductase | K00367 | *narB* |
|  | *narC* | Cytochrome b-561 | K15879 | *narC* |
|  | *nasA* | Assimilatory nitrate reductase catalytic subunit | K00372 | *nasC, nasA* |
|  | *nasB* | Assimilatory nitrate reductase electron transfer subunit | K00360 | *nasB* |
|  | *nirA* | Ferredoxin-nitrite reductase | K00366 | *nirA* |
|  | *NR* | Nitrate reductase (NAD(P)H) | K10534 | *NR* |
| Organic degradation and synthesis (ODAS) | *gdh_K00260* | Glutamate dehydrogenase | K00260 | *gudB, rocG* |
|  | *gdh_K00261* | Glutamate dehydrogenase (NAD(P)+) | K00261 | *GLUD1_2, gdhA* |
|  | *gdh_K00262* | Glutamate dehydrogenase (NADP+) | K00262 | *E1.4.1.4, gdhA* |
|  | *gdh_K15371* | Glutamate dehydrogenase | K15371 | *GDH2* |
|  | *ansB* | Glutamin-(asparagin-)ase | K05597 | *aspQ, ansB, ansA* |
|  | *glsA* | Glutaminase | K01425 | *glsA, GLS* |
|  | *ureA* | Urease subunit gamma | K01430 | *ureA* |
|  | *ureB* | Urease subunit beta | K01429 | *ureB* |
|  | *ureC* | Urease subunit alpha | K01428 | *ureC* |
|  | *gs_K00264* | Glutamate synthase (NADPH/NADH) | K00264 | *GLT1* |
|  | *gs_K00265* | Glutamate synthase (NADPH/NADH) large chain | K00265 | *gltB* |
|  | *gs_K00266* | Glutamate synthase (NADPH/NADH) small chain | K00266 | *gltD* |
|  | *gs_K00284* | Glutamate synthase (ferredoxin) | K00284 | *GLU, gltS* |
|  | *asnB* | Asparagine synthase (glutamine-hydrolysing) | K01953 | *asnB, ASNS* |
|  | *glnA* | Glutamine synthetase | K01915 | *glnA, GLUL* |
|  | *nao* | Nitroalkane oxidase | K19823 | *NAO* |
|  | *nmo* | Nitronate monooxygenase | K00459 | *ncd2, npd* |
| Nitrogen fixation | *anfG* | Nitrogenase delta subunit | K00531 | *anfG* |
|  | *nifD* | Nitrogenase molybdenum-iron protein alpha chain | K02586 | *nifD* |
|  | *nifH* | Nitrogenase iron protein NifH | K02588 | *nifH* |
|  | *nifK* | Nitrogenase molybdenum-iron protein beta chain | K02591 | *nifK* |
|  | *nifW* | Nitrogenase-stabilizing/protective protein | K02595 | *nifW* |
| Nitrification | *amoA_A* | Ammonia monooxygenase subunit A (archaea) | K10944 | *pmoA-amoA* |
|  | *amoA_B* | Ammonia monooxygenase subunit B (archaea) | K10944 | *pmoA-amoA* |
|  | *amoB_A* | Ammonia monooxygenase subunit C (archaea) | K10945 | *pmoB-amoB* |
|  | *amoB_B* | Ammonia monooxygenase subunit A (bacteria) | K10945 | *pmoB-amoB* |
|  | *amoC_A* | Ammonia monooxygenase subunit B (bacteria) | K10946 | *pmoC-amoC* |
|  | *amoC_B* | Ammonia monooxygenase subunit C (bacteria) | K10946 | *pmoC-amoC* |
|  | *hao* | Hydroxylamine dehydrogenase | K10535 | *hao* |
|  | *nxrA* | Nitrite oxidoreductase, alpha subunit | K00370 | *narG, narZ, nxrA* |
|  | *nxrB* | Nitrite oxidoreductase, beta subunit | K00371 | *narH, narY, nxrB* |
| anammox | *hdh* | Hydrazine oxidoreductase | K20935 | *hdh* |
|  | *hzsA* | Hydrazine synthase subunit A | K20932 | *K20932* |
|  | *hzsB* | Hydrazine synthase subunit B | K20933 | *K20933* |
|  | *hzsC* | Hydrazine synthase subunit C | K20934 | *K20934* |
|  | *hzo* | Hydrazine dehydrogenase | K20935 | *hdh* |
| Others | *hcp* | Hydroxylamine reductase | K05601 | *hcp* |
|  | *pmoA* | Particulate methane monooxygenase subunit A | K10944 | *pmoA-amoA* |
|  | *pmoB* | Particulate methane monooxygenase subunit B | K10945 | *pmoB-amoB* |
|  | *pmoC* | Particulate methane monooxygenase subunit C | K10946 | *pmoC-amoC* |

*The genes that perform nitrate reduction to nitrite in the DNRA pathway and denitrification pathway are equivalent, and they are shown in duplicate in the table for the 2 pathways.

**Supplementary Table S6** Global basic information for random forest regression models.

| **Random Forest Regression Model** | **predictor variable** | **response variable** | **% Var explained** | **cross-validation**  **R^2^** | **p value** |
| --- | --- | --- | --- | --- | --- |
| DEN1^*a^ | 132 MAGs annotated to contain functional genes for denitrification (no nosZ) at D7 | N_2_O AUC at D7  (Fig. 1a) ^*b^ | 81.56% | 85.40% | < 0.001 |
| DEN2^*a^ | 241 MAGs annotated to contain functional genes for denitrification (with nosZ) at D7 | total denitrification at D7  (Fig. 1a, 1b)^*c^ | 82.42% | 73.70% | < 0.001 |
| DEN3^*a^ | 132 MAGs annotated to contain functional genes for denitrification (no nosZ) at M12 | N_2_O AUC at M12  (Fig. 1d) ^*b^ | 57.80% | 63.70% | < 0.001 |
| DEN4^*a^ | 241 MAGs annotated to contain functional genes for denitrification (with nosZ) at M12 | total denitrification at M12 (Fig. 1d, 1e) ^*c^ | 82.95% | 80.90% | < 0.001 |
| N_fixation | 79 MAGs annotated to contain functional genes for nitrogen fixation | nitrogen fixation rate (Fig. 1i) | 75.20% | 80.60% | < 0.001 |
| product_NH4 | 461 MAGs annotated to contain functional genes for catalytic ammonium production | ammonium content  (Fig. 1g) | 71.48% | 73.20% | < 0.001 |

**a**: DEN1-DEN4 were the four random forest regression models related to denitrification function. Since denitrification-related parameters were mainly measured dynamically at the initial stage of incubation and after 1 year of incubation, the random forest regression models were constructed separately according to the time points (D7, M12) and corresponding denitrification gas parameters.

**b**: “N_2_O AUC” refers to the area under the curve of the dynamics of N_2_O content in each group over a fixed time range.

**c**: “total denitrification” refers to the sum of the N_2_O and N_2_ gas content produced by each group at a fixed timepoint (50 h), which represents the total amount of externally added nitrate converted by the denitrification pathway.

**Supplementary Table S7** The important MAGs and their significance in 6 random forest regression models.

|  | %IncMSE | pval |  | %IncMSE | pval |  | %IncMSE | pval |
| --- | --- | --- | --- | --- | --- | --- | --- | --- |
| **product_NH4** | | | **N_fixation** | | | **DEN1** | | |
| MAG603 | 7.34 | ** | MAG92 | 7.24 | ** | MAG587 | 8.31 | ** |
| MAG174 | 6.40 | ** | MAG137 | 7.93 | ** | MAG177 | 6.62 | ** |
| MAG521 | 10.90 | ** | MAG520 | 6.10 | * | MAG53 | 7.65 | ** |
| MAG186 | 6.67 | ** | MAG516 | 7.33 | ** | **DEN2** | | |
| MAG495 | 11.50 | ** | MAG638 | 7.76 | ** | MAG609 | 3.72 | * |
| MAG539 | 7.26 | ** | MAG651 | 8.08 | ** | MAG641 | 3.58 | * |
| MAG296 | 6.66 | * | MAG434 | 6.69 | ** | MAG256 | 3.72 | * |
| MAG331 | 6.84 | * | MAG369 | 7.57 | ** | MAG93 | 3.47 | * |
| MAG500 | 11.54 | ** | MAG438 | 7.67 | ** | MAG645 | 3.68 | * |
| MAG106 | 11.44 | ** | MAG112 | 8.52 | * | MAG610 | 3.26 | * |
| MAG259 | 7.51 | * | MAG483 | 8.27 | ** | MAG40 | 3.01 | * |
| MAG160 | 10.89 | ** | MAG340 | 7.83 | * | MAG101 | 2.99 | * |
| MAG184 | 14.05 | ** | MAG193 | 8.20 | * | **DEN3** | | |
| MAG170 | 11.59 | ** |  |  |  | MAG27 | 4.54 | ** |
| MAG11 | 10.98 | ** |  |  |  | MAG526 | 4.30 | * |
| MAG561 | 7.47 | ** |  |  |  | MAG455 | 3.49 | * |
| MAG486 | 6.57 | ** |  |  |  | MAG502 | 4.19 | * |
| MAG10 | 14.49 | ** |  |  |  | MAG16 | 3.49 | ** |
| MAG643 | 14.25 | ** |  |  |  | MAG26 | 3.70 | * |
| MAG14 | 14.90 | ** |  |  |  | MAG581 | 5.12 | ** |
| MAG143 | 14.90 | ** |  |  |  | **DEN4** | | |
| MAG556 | 13.56 | ** |  |  |  | MAG172 | 4.15 | ** |
| MAG222 | 7.40 | ** |  |  |  | MAG171 | 4.05 | ** |
| MAG504 | 14.55 | ** |  |  |  | MAG103 | 3.85 | ** |
| MAG532 | 14.57 | ** |  |  |  | MAG573 | 2.68 | * |
| MAG541 | 11.05 | ** |  |  |  | MAG598 | 3.84 | * |
|  |  |  |  |  |  | MAG192 | 3.75 | ** |
|  |  |  |  |  |  | MAG536 | 3.26 | ** |

**Supplementary Table S8** Two-way ANOVA statistics examing effects of time, treatment (ACN, ACM, ACP, ACS), and their interaction on nitrogen parameters (n=3 per time and treatment combination).

| **Parameter** |  | **Time** | **Treatment** | **Time*Treatment** |
| --- | --- | --- | --- | --- |
| **N_2_O index** | **F** | 8.519 | 177 | 25.1 |
|  | **P** | P<0.0001 | P<0.0001 | P=0.0013 |
|  | **sign.** | **** | **** | *** |
| **NH_4_^+^-N**^a^ | **F** | 306.3 | 136.3 | 10.59 |
|  | **P** | P<0.0001 | P<0.0001 | P<0.0001 |
|  | **sign.** | **** | **** | **** |
| **N_2_ accumulation** | **F** | 211.2 | 241.8 | 23.31 |
|  | **P** | P<0.0001 | P<0.0001 | P<0.0001 |
|  | **sign.** | **** | **** | **** |
| **Estimated N_2_ fixation** | **F** | 125.8 | 136.5 | 2.978 |
|  | **P** | P=0.0891 | P<0.0001 | P<0.0001 |
|  | **sign.** |  | **** | **** |

**a**: **on log-transformed data**

**Supplementary Table S9** Two-way ANOVA statistics examing effects of time (7 days, 6 months and 12 months of incubation), treatment (ACN, ACM, ACP, ACS), and their interaction on nitrogen functional genes (n=3 per time and treatment combination).

| **Parameter** |  | **Time** | **Treatment** | **Time*Treatment** |
| --- | --- | --- | --- | --- |
| ***ureC*** | **F** | 234.2 | 93.36 | 14.85 |
|  | **P** | P<0.0001 | P<0.0001 | P<0.0001 |
|  | **sign.** | **** | **** | **** |
| ***gdh_K15371*** | **F** | 650.5 | 62 | 32..05 |
|  | **P** | P<0.0001 | P<0.0001 | P<0.0001 |
|  | **sign.** | **** | **** | **** |
| ***gdh_K00262*** | **F** | 54.61 | 100.6 | 7.46 |
|  | **P** | P=0.0001 | P<0.0001 | P<0.0001 |
|  | **sign.** | *** | **** | **** |
| ***gdh_K00261*** | **F** | 64.84 | 134.8 | 13.24 |
|  | **P** | P<0.0001 | P<0.0001 | P<0.0001 |
|  | **sign.** | **** | **** | **** |
| ***glsA*** | **F** | 160.9 | 93.61 | 13.49 |
|  | **P** | P<0.0001 | P<0.0001 | P<0.0001 |
|  | **sign.** | **** | **** | **** |
| ***ansB*** | **F** | 1.96 | 16.06 | 3.555 |
|  | **P** | P<0.0001 | P=0.1627 | P=0.0116 |
|  | **sign.** | **** |  | * |
| ***nirA*** | **F** | 158.7 | 40.19 | 8.736 |
|  | **P** | P<0.0001 | P<0.0001 | P<0.0001 |
|  | **sign.** | **** | **** | **** |
| ***nrfC*** | **F** | 9.46 | 1.979 | 10.89 |
|  | **P** | P=0.1441 | P=0.0009 | P<0.0001 |
|  | **sign.** |  | **** | **** |
| ***nrfA*** | **F** | 1.724 | 15.46 | 3.194 |
|  | **P** | P<0.0001 | P=0.1997 | P=0.019 |
|  | **sign.** | **** |  | * |
| ***nirD*** | **F** | 75.64 | 33.46 | 4.508 |
|  | **P** | P<0.0001 | P<0.0001 | P=0.0034 |
|  | **sign.** | **** | **** | ** |
| ***nirB*** | **F** | 218.3 | 34.08 | 7.067 |
|  | **P** | P<0.0001 | P<0.0001 | P=0.0002 |
|  | **sign.** | **** | **** | *** |
| ***nosZ*** | **F** | 65.98 | 64.08 | 18.34 |
|  | **P** | P<0.0001 | P<0.0001 | P<0.0001 |
|  | **sign.** | **** | **** | **** |
| ***norB*** | **F** | 24.92 | 43.36 | 6.713 |
|  | **P** | P<0.0001 | P<0.0001 | P=0.0003 |
|  | **sign.** | **** | **** | *** |
| ***nirS*** | **F** | 6.655 | 4.636 | 7.769 |
|  | **P** | P=0.005 | P=0.0108 | P=0.0001 |
|  | **sign.** | ** | * | *** |
| ***nirK*** | **F** | 211.5 | 120.6 | 33.6 |
|  | **P** | P<0.0001 | P<0.0001 | P<0.0001 |
|  | **sign.** | **** | **** | **** |
| ***narG*** | **F** | 30.62 | 30.42 | 18.37 |
|  | **P** | P<0.0001 | P<0.0001 | P<0.0001 |
|  | **sign.** | **** | **** | **** |
| ***napA*** | **F** | 41.16 | 20.69 | 12.53 |
|  | **P** | P<0.0001 | P<0.0001 | P<0.0001 |
|  | **sign.** | **** | **** | **** |
| ***nifK*** | **F** | 121.5 | 216.8 | 38.1 |
|  | **P** | P<0.0001 | P<0.0001 | P<0.0001 |
|  | **sign.** | **** | **** | **** |
| ***nifH*** | **F** | 393.9 | 441 | 110.3 |
|  | **P** | P<0.0001 | P<0.0001 | P<0.0001 |
|  | **sign.** | **** | **** | **** |
| ***nifD*** | **F** | 72.77 | 185.9 | 26.43 |
|  | **P** | P<0.0001 | P<0.0001 | P<0.0001 |
|  | **sign.** | **** | **** | **** |

**Supplementary Table S10.** Repeated Measures Two-way ANOVA statistics examining effects of time, treatment (ACN, ACM, ACP, ACS), and their interaction on denitrification gas dynamics (n=3 per time and treatment combination). Geisser-Greenhouse correction was applied to adjust for violations of sphericity.

| **Parameter** |  | **Time** | **Treatment** | **Time***  **Treatment** | **Geisser-Greenhouse**  **є** |
| --- | --- | --- | --- | --- | --- |
| **N_2_O**  **intial** | **F** | 108 | 235.4 | 32.45 | 0.1455 |
|  | **P** | P<0.0001 | P<0.0001 | P<0.0001 |  |
|  | **sign.** | **** | **** | **** |  |
| **N_2_**  **intial** | **F** | 547.8 | 127.4 | 85.1 | 0.1506 |
|  | **P** | P<0.0001 | P<0.0001 | P<0.0001 |  |
|  | **sign.** | **** | **** | **** |  |
| **NO**  **intial** | **F** | 342.5 | 186 | 143.4 | 0.0886 |
|  | **P** | P<0.0001 | P<0.0001 | P<0.0001 |  |
|  | **sign.** | **** | **** | **** |  |
| **N_2_O**  **after 1 year** | **F** | 14.69 | 12.9 | 10.05 | 0.07286 |
|  | **P** | P<0.0001 | P=0.0047 | P=0.002 |  |
|  | **sign.** | **** | ** | ** |  |
| **N_2_**  **after 1 year** | **F** | 309.4 | 22.73 | 17.9 | 0.08171 |
|  | **P** | P<0.0001 | P=0.0003 | P<0.0001 |  |
|  | **sign.** | **** | *** | **** |  |

**Explanation:** The Geisser-Greenhouse ε values in this table indicate the degree of sphericity assumption violation. Lower ε values suggest a greater departure from sphericity, thus requiring this correction. For instance, a low ε value (e.g., 0.07286 for N₂O measured after one year) led to the adjustment of degrees of freedom using the Geisser-Greenhouse correction to ensure the reliability of p-values. This correction is a standard procedure in repeated measures designs when the sphericity assumption is violated, making the reported p-values more conservative and statistically robust.

**Supplementary Table S11.** PERMANOVA results for different datasets (e.g., 16S ASV, species-level abundance, KEGG pathways, N-cycling genes, and CAZymes), showing the statistical significance and effect sizes of treatment, sampling time, and their interaction on microbial and functional community compositions.

| **All_sample**  **16S_ASV** | **Factor** | **Df** | **F.Model** | **R2** | **Pr(>F)** |
| --- | --- | --- | --- | --- | --- |
|  | groups | 4 | 16.7773 | 0.35498 | 0.001*** |
|  | sampletime | 4 | 9.7141 | 0.20553 | 0.001*** |
|  | groups:sampletime | 12 | 3.4238 | 0.21733 | 0.001*** |
|  | Residuals | 42 | - | 0.22216 | - |
|  | Total | 62 | - | 1 | - |
| **meta_species** | **Factor** | **Df** | **F.Model** | **R2** | **Pr(>F)** |
|  | groups | 4 | 56.755 | 0.51711 | 0.001*** |
|  | sampletime | 2 | 64.193 | 0.29244 | 0.001*** |
|  | groups:sampletime | 6 | 9.602 | 0.13123 | 0.001*** |
|  | Residuals | 26 | - | 0.05922 | - |
|  | Total | 38 | - | 1 | - |
| **meta_kegg**  **all_KO** | **Factor** | **Df** | **F.Model** | **R2** | **Pr(>F)** |
|  | groups | 4 | 37.095 | 0.33432 | 0.001*** |
|  | sampletime | 2 | 101.833 | 0.45889 | 0.001*** |
|  | groups:sampletime | 6 | 10.963 | 0.1482 | 0.001*** |
|  | Residuals | 26 | - | 0.05858 | - |
|  | Total | 38 | - | 1 | - |
| **meta_NcycDB** | **Factor** | **Df** | **F.Model** | **R2** | **Pr(>F)** |
|  | groups | 4 | 50.392 | 0.37874 | 0.001*** |
|  | sampletime | 2 | 115.986 | 0.43587 | 0.001*** |
|  | groups:sampletime | 6 | 12.11 | 0.13653 | 0.001*** |
|  | Residuals | 26 | - | 0.04885 | - |
|  | Total | 38 | - | 1 | - |
| **meta_CAZymes** | **Factor** | **Df** | **F.Model** | **R2** | **Pr(>F)** |
|  | groups | 4 | 40.288 | 0.39541 | 0.001*** |
|  | sampletime | 2 | 83.024 | 0.40743 | 0.001*** |
|  | groups:sampletime | 6 | 9.058 | 0.13336 | 0.001*** |
|  | Residuals | 26 | - | 0.0638 | - |
|  | Total | 38 | - | 1 | - |

The table shows the degrees of freedom (Df), F-statistics (F.Model), R² values, and p-values (Pr(>F)) for the effects of treatment (groups), sampling time, and their interaction on microbial and functional community composition. Statistical significance is indicated by asterisks: p < 0.05 *, p < 0.01 **, and p < 0.001 ***. R² values represent the proportion of variance explained by each factor, and higher values indicate greater effect sizes.

**Supplementary Table S12**. Sequencing and Bioinformatics Parameters.

| Analysis Step | Software/Tool | Parameters |
| --- | --- | --- |
| Adapter and Primer Trimming | QIIME2 Cutadapt | --p-front-f CCTACGGGNGGCWGCAG, --p-front-r GACTACHVGGGTATCTAATCC |
| Denoising and ASV merging | QIIME2 DADA2 | --p-trim-left-f 0, --p-trim-left-r 0, --p-trunc-len-f 256, --p-trunc-len-r 202 |
| Alpha rarefaction analysis | QIIME2 | max-depth=43000, min-depth=1000, steps=5000 |
| Metagenome-assembled genome (MAG) binning | MetaBAT2 | min contig length=1500, threads=16 |
| Bins quality filtering | CheckM | workflow=lineage_wf, threads=20, file extension=fa, nucleotide-level (--nt), tabular output (--tab_table) |
| Bins dereplication | dRep | similarity threshold (-sa)=0.99, contamination threshold (-nc)=0.10, completeness threshold (-comp)=50, contamination max (-con)=10 |
| Read mapping and quantification | CoverM | -m relative abundance, -t 16 |
| Taxonomic annotation of non-redundant MAGs | GTDB-Tk | gtdbtk classify_wf --genome_dir <my_genomes> --out_dir <output_dir> -x fa |
| Phylogenetic tree construction | Phylophlan v.3.0 | --diversity high -d phylophlan -t a --accurate -f drep0.99_470bins_config_n_test.cfg |
| Nitrogen cycling gene annotation | NcycDB | E-value 10^-5^ |
| Carbohydrate degradation-related gene annotation | CAZy | default E-value |
| Functional annotation of MAGs | KEGG (Kofam_scan) | -E 0.0001 -f mapper |
| KEGG pathway completeness analysis | KEGGDecoder | -i kofam_mapper.txt -o function.list -v static |

**Note**: Figures were generated using a combination of software tools: bar and stacked bar charts were created in Prism, PCoA plots, heatmaps, and bubble charts were generated in R (ggplot2 package), and phylogenetic trees were visualized using the iTOL web-based tool.

**Supplementary Table S13**. Kruskal-Wallis Test Results for Phylum-Level Differences Based on 16S rRNA V3-V4 Region High-Throughput Sequencing Analysis: Comparison of Within-Group Temporal Variations and Between-Group Differences at Each Time Point (See Fig. 2b for Phylum-Level Stacked Bar Plots).

| **Phylum** | **Group Comparison**  **(Same TimePoint)** | | | **Time Comparison**  **(Same Group)** | | |
| --- | --- | --- | --- | --- | --- | --- |
|  | **TimePoint** | **p_value** | **Sign.** | **Group** | **p_value** | **Sign.** |
| Acidobacteriota | D7 | 0.0237 | * | ACN | 0.1991 | ns |
|  | M3 | 0.0237 | * | ACM | 0.0241 | * |
|  | M6 | 0.1681 | ns | ACP | 0.0347 | * |
|  | M9 | 0.0156 | * | ACS | 0.0262 | * |
|  | M12 | 0.0156 | * |  |  |  |
| Actinobacteriota | D7 | 0.0770 | ns | ACN | 0.1032 | ns |
|  | M3 | 0.0188 | * | ACM | 0.0174 | * |
|  | M6 | 0.0627 | ns | ACP | 0.0102 | * |
|  | M9 | 0.0237 | * | ACS | 0.0102 | * |
|  | M12 | 0.0232 | * |  |  |  |
| Armatimonadota | D7 | 0.3693 | ns | ACN | 0.6581 | ns |
|  | M3 | 0.1079 | ns | ACM | 0.0585 | ns |
|  | M6 | 0.0375 | * | ACP | 0.0203 | * |
|  | M9 | 0.0656 | ns | ACS | 0.0148 | * |
|  | M12 | 0.1349 | ns |  |  |  |
| Bacteroidota | D7 | 0.0824 | ns | ACN | 0.1491 | ns |
|  | M3 | 0.0156 | * | ACM | 0.0181 | * |
|  | M6 | 0.0752 | ns | ACP | 0.0121 | * |
|  | M9 | 0.0749 | ns | ACS | 0.0091 | ** |
|  | M12 | 0.0329 | * |  |  |  |
| Chloroflexi | D7 | 0.0216 | * | ACN | 0.0672 | ns |
|  | M3 | 0.0862 | ns | ACM | 0.0293 | * |
|  | M6 | 0.1349 | ns | ACP | 0.0144 | * |
|  | M9 | 0.0324 | * | ACS | 0.0090 | ** |
|  | M12 | 0.0862 | ns |  |  |  |
| Desulfobacterota | D7 | 0.3693 | ns | ACN | 0.1537 | ns |
|  | M3 | 0.0249 | * | ACM | 0.1074 | ns |
|  | M6 | 0.0378 | * | ACP | 0.0091 | ** |
|  | M9 | 0.0156 | * | ACS | 0.0385 | * |
|  | M12 | 0.0156 | * |  |  |  |
| Fibrobacterota | D7 | 0.2239 | ns | ACN | 0.5793 | ns |
|  | M3 | 0.0345 | * | ACM | 0.0113 | * |
|  | M6 | 0.0559 | ns | ACP | 0.0154 | * |
|  | M9 | 0.0213 | * | ACS | 0.0151 | * |
|  | M12 | 0.0245 | * |  |  |  |
| Firmicutes | D7 | 0.0156 | * | ACN | 0.0471 | * |
|  | M3 | 0.0307 | * | ACM | 0.0144 | * |
|  | M6 | 0.0237 | * | ACP | 0.0979 | ns |
|  | M9 | 0.0156 | * | ACS | 0.0116 | * |
|  | M12 | 0.0156 | * |  |  |  |
| Gemmatimonadota | D7 | 0.0284 | * | ACN | 0.0262 | * |
|  | M3 | 0.0245 | * | ACM | 0.0277 | * |
|  | M6 | 0.0415 | * | ACP | 0.0156 | * |
|  | M9 | 0.0752 | ns | ACS | 0.0285 | * |
|  | M12 | 0.0237 | * |  |  |  |
| Methylomirabilota | D7 | 0.0499 | * | ACN | 0.1429 | ns |
|  | M3 | 0.0973 | ns | ACM | 0.0280 | * |
|  | M6 | 0.0415 | * | ACP | 0.0823 | ns |
|  | M9 | 0.0534 | ns | ACS | 0.4017 | ns |
|  | M12 | 0.0315 | * |  |  |  |
| Myxococcota | D7 | 0.0216 | * | ACN | 0.0388 | * |
|  | M3 | 0.0770 | ns | ACM | 0.0224 | * |
|  | M6 | 0.0484 | * | ACP | 0.2548 | ns |
|  | M9 | 0.0770 | ns | ACS | 0.0408 | * |
|  | M12 | 0.3093 | ns |  |  |  |
| Nitrospirota | D7 | 0.1079 | ns | ACN | 0.1967 | ns |
|  | M3 | 0.0378 | * | ACM | 0.0156 | * |
|  | M6 | 0.1009 | ns | ACP | 0.0111 | * |
|  | M9 | 0.0230 | * | ACS | 0.0202 | * |
|  | M12 | 0.0307 | * |  |  |  |
| Planctomycetota | D7 | 0.0245 | * | ACN | 0.0698 | ns |
|  | M3 | 0.0185 | * | ACM | 0.0928 | ns |
|  | M6 | 0.0922 | ns | ACP | 0.0357 | * |
|  | M9 | 0.0188 | * | ACS | 0.0781 | ns |
|  | M12 | 0.0216 | * |  |  |  |
| Proteobacteria | D7 | 0.0237 | * | ACN | 0.1648 | ns |
|  | M3 | 0.0188 | * | ACM | 0.0111 | * |
|  | M6 | 0.0261 | * | ACP | 0.0166 | * |
|  | M9 | 0.0156 | * | ACS | 0.0212 | * |
|  | M12 | 0.0752 | ns |  |  |  |
| Spirochaetota | D7 | NA | - | ACN | NA | - |
|  | M3 | 0.0879 | ns | ACM | 0.0134 | * |
|  | M6 | 0.0474 | * | ACP | 0.0154 | * |
|  | M9 | 0.0223 | * | ACS | 0.0121 | * |
|  | M12 | 0.0145 | * |  |  |  |

**1.2 Supplementary Figures**


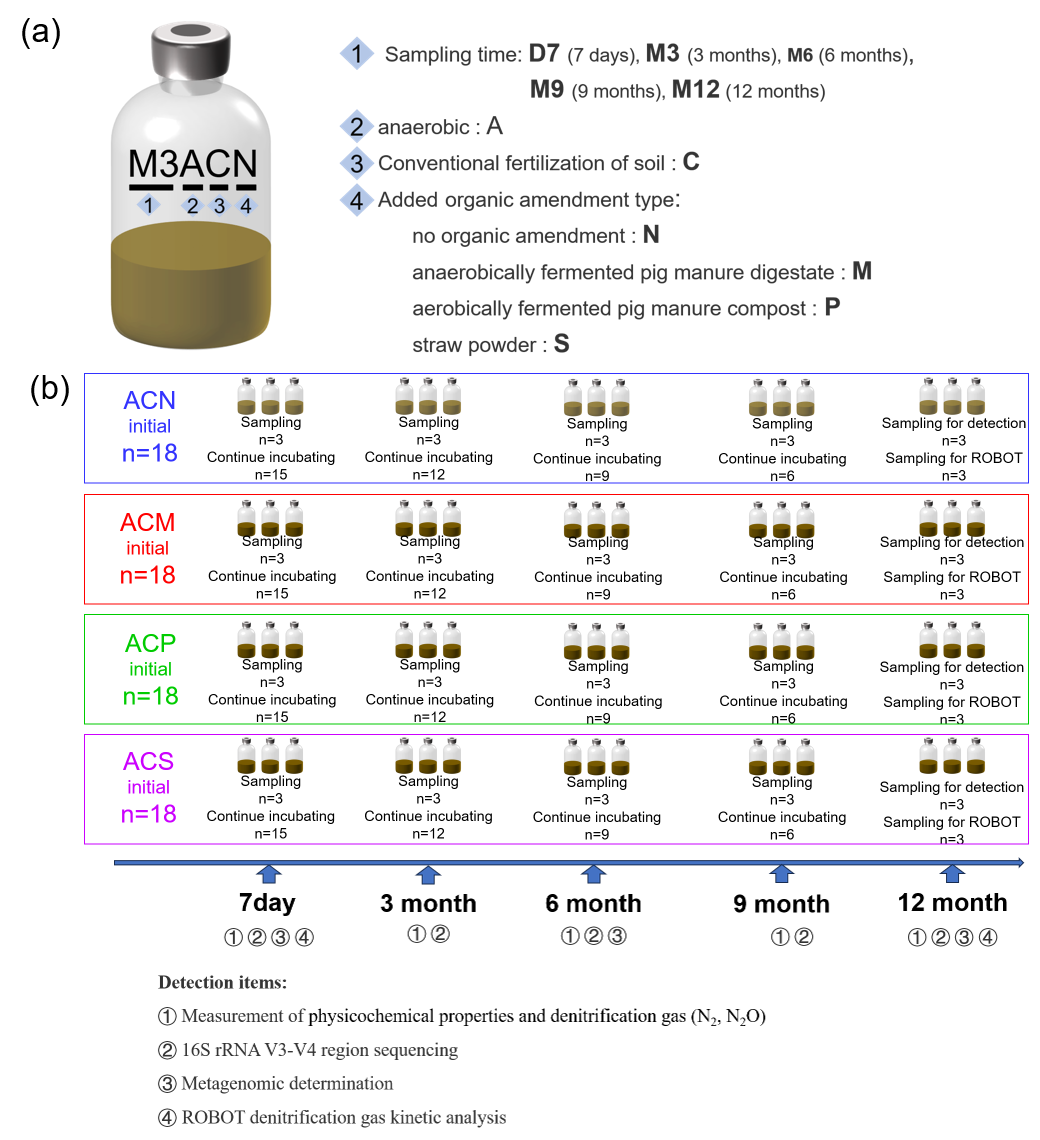


**Supplementary Figure S1** Schematic diagram of experimental design and experimental group abbreviations. (A) The treatments of experiment and naming scheme. (B) Schematic diagram of experimental design


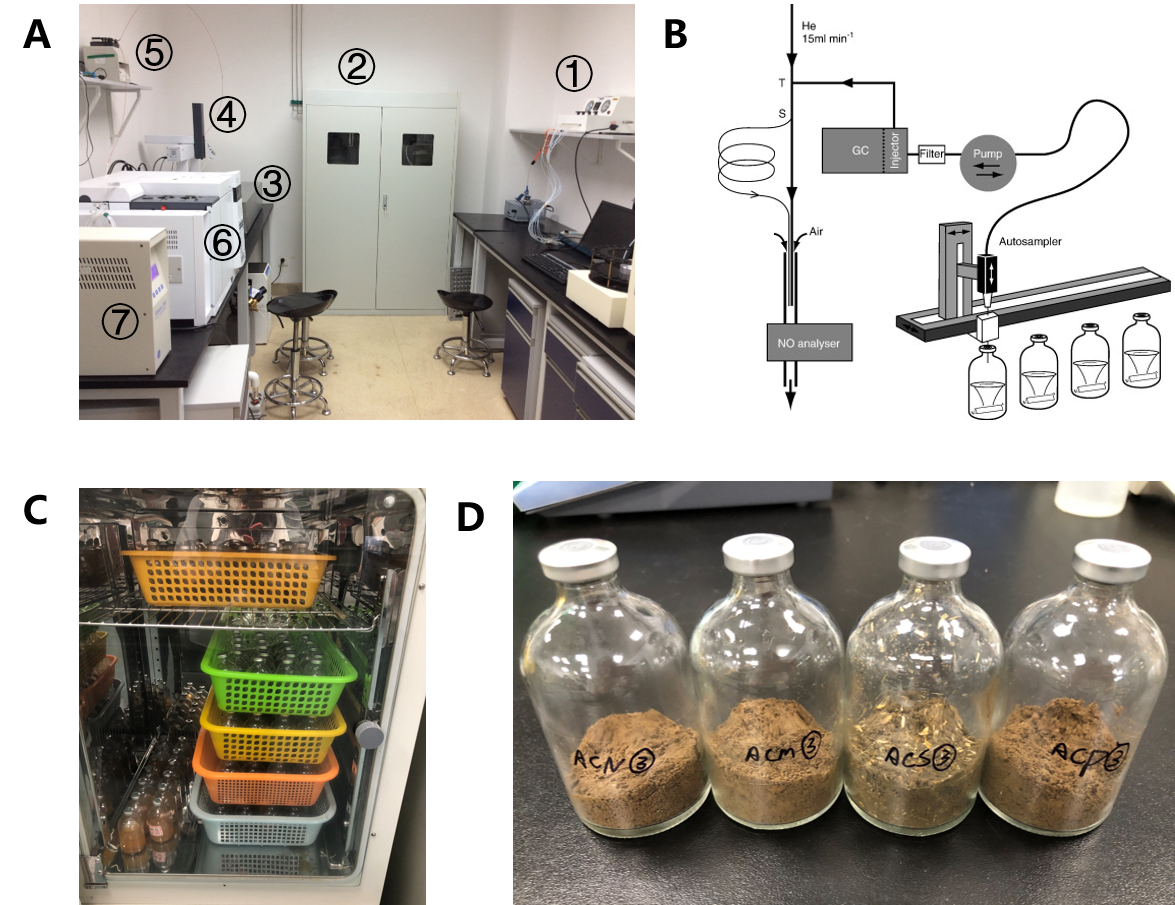


**Supplementary Figure S2** Schematic diagram of the soil microcosm in anaerobic long-term cultivation and the ROBOT system. ROBOT is an automated system designed for continuous monitoring of gas concentrations within a cultivation system. The system can detect six gases: O₂, CO₂, N₂, NO, N₂O, and CH₄.

(A): ROBOT system used in this study. ① Semi-automatic gas replacement unit, ② Gas cylinder cabinet, ③ Sample cultivation and fixation unit, ④ Automated sampling robotic arm, ⑤ Peristaltic pump, ⑥ Gas chromatograph (GC-7890A), ⑦ NO detection. (B): a simplified version of the ROBOT system from the original publication (Molstad et al., 2007). Reproduced with permission from Molstad et al., 2007, *J. Microbiol. Methods* 68, 439–446 (Elsevier). (C): long-term cultivation in temperature-controlled incubator. (D): the serum bottles used for cultivation. The sampling needle from the ROBOT system collects gas from the rubber septum at the area where the aluminum cap is hollow.

**ROBOT System Overview**: The system can be understood as a series of interconnected devices. These include: ① Semi-automatic gas replacement unit: This unit is used for pre-treatment, replacing the gases inside the 120 ml serum bottle with high-purity helium to create anaerobic conditions. ② Gas cylinder cabinet: This is connected to the entire system, supplying the necessary gases for various components, including the gas replacement unit. ③ Sample cultivation and fixation unit: This includes a circulating water bath and other components for maintaining sample stability. ④ Automated sampling robotic arm: The system is controlled by Python code to manipulate the robotic arm, allowing for slight variations in sampling positions to minimize damage to the same area of the rubber septum, thus maintaining anaerobic conditions. ⑤ Peristaltic pump: This pump provides the necessary power for gas extraction during the sampling process. ⑥ Gas chromatograph (GC-7890A) : Used to detect all gases except NO. ⑦ NO detection: Specifically used to monitor NO.

**Operating Procedure:**

1. Sample Pre-treatment:

Before starting the experiment, all samples are weighed and added under sterile conditions in a laminar flow hood. After sealing the samples, they are transferred to the ROBOT room. The gas replacement system is then used to replace the air in each sample bottle with helium. This is done by inserting a fine needle connected to the gas replacement device into the sample bottle, following a procedure of 35 seconds of vacuuming and 10 seconds of helium injection, repeated at least 4 times. After the final injection, the needle is removed, and the bottle remains at positive pressure. The pressure is then balanced by inserting a 10ml syringe containing ddH₂O, which allows any excess helium to slowly escape in bubble form. Once the bubbling stops, the syringe is removed, and the bottle is equilibrated to atmospheric pressure. After this procedure, the samples are transferred to a temperature-controlled incubator for long-term cultivation (Figure C). Samples reserved for initial-stage denitrification gas dynamics analysis are set aside for further steps.

1. System Setup:

The pre-treated sample bottles are fixed in the temperature-controlled water bath to ensure accurate positioning for sampling. Each batch includes air bottles as positive controls and helium bottles as negative controls, ensuring that the entire sampling system operates without gas leakage.

1. Sampling Procedure:

The sampling program is coded in Python. Sampling duration and tubing flushing times are set accordingly. Each sample is collected for 300 seconds, and the extracted gases are replaced with helium. Samples are collected approximately every 7 minutes, and the sampling system operates in a fixed order. Each sample has multiple sampling points, with a fixed interval of approximately 4 hours between each pair of sampling points

1. Data Processing:

Data obtained after sampling are pre-processed using a fixed code. The signal strength data is imported into a calculation sheet available on Lars Molstad's website (https://www.nmbu.no/en/research/groups/nmbu-nitrogen-group), where specific gas concentrations are calculated.





**Supplementary Figure S3** The proportion of carbon fractions in each group after the addition of organic matter.

Chemical structure spectra of soil organic carbon in each group after the addition of organic amendment determined by ^13^C NMR (Fig. S3A). The composition of the carbon fractions was first chemically shifted to divide the eight functional groups (Fig. S3C), of which four groups had a higher proportion of 65-94 ppm alkyne C alkyne carbon, probably the C2-C6 signal in the aminosaccharide or pyranoside structures, and this ACM and ACS groups had the highest signal intensities at the chemical shifts. 162-188 ppm of carboxyl and amide carbon COO/N-C= O was also higher in the ACP group than in the other three groups. 0-45 ppm of alkyl carbon alkyl C was also higher in the ACP group than in the other three groups. The ACM and ACS groups also had a higher proportion of dioxyalkyl carbons than the remaining three groups, with this functional group originating from hemicellulose. Reference was made to the literature by Huang et al. where O-alkyl, O-aryl, and carboxy C were calculated as labile C and alkyl, N-alkyl/methoxyl, and aryl C as recalcitrant C (Fig. S3B). The results showed that the ACM and ACS groups had a relatively higher content of recalcitrant carbon, while the ACP and ACN groups contained mainly labile carbon.


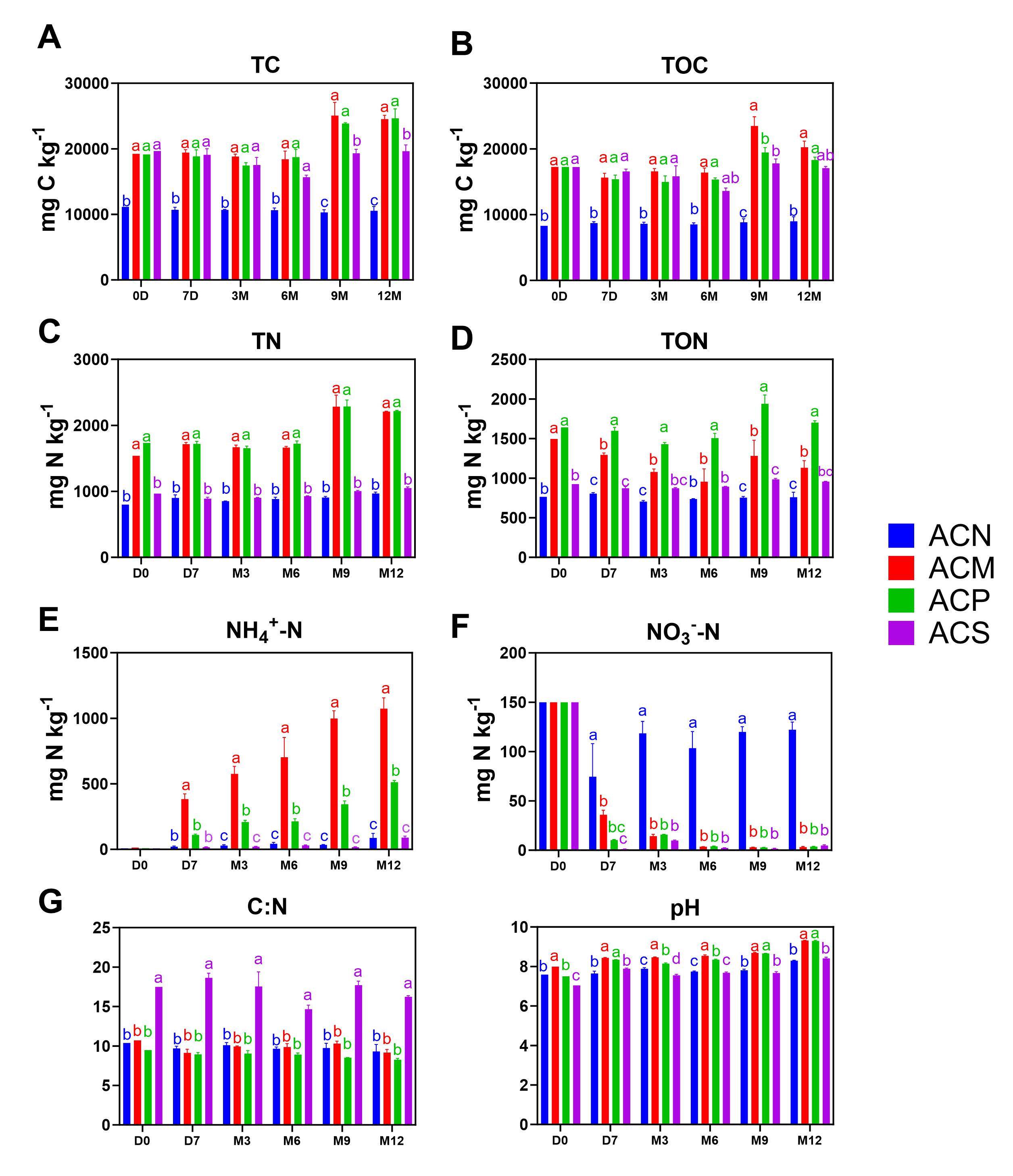


**Supplementary Figure S4** Physical and chemical parameters of each group during incubation.

The addition of the three organic amendments significantly increased soil TC, and TOC content. Although the TC and TOC contents decreased with longer incubation time, the addition of organic amendments still caused significant carbon sequestration. Organic amendment addition accelerated the utilization of nitrate by soil microbial community. Digestate and compost had little effect on soil C:N, whereas straw addition significantly increased soil carbon to nitrogen ratio.


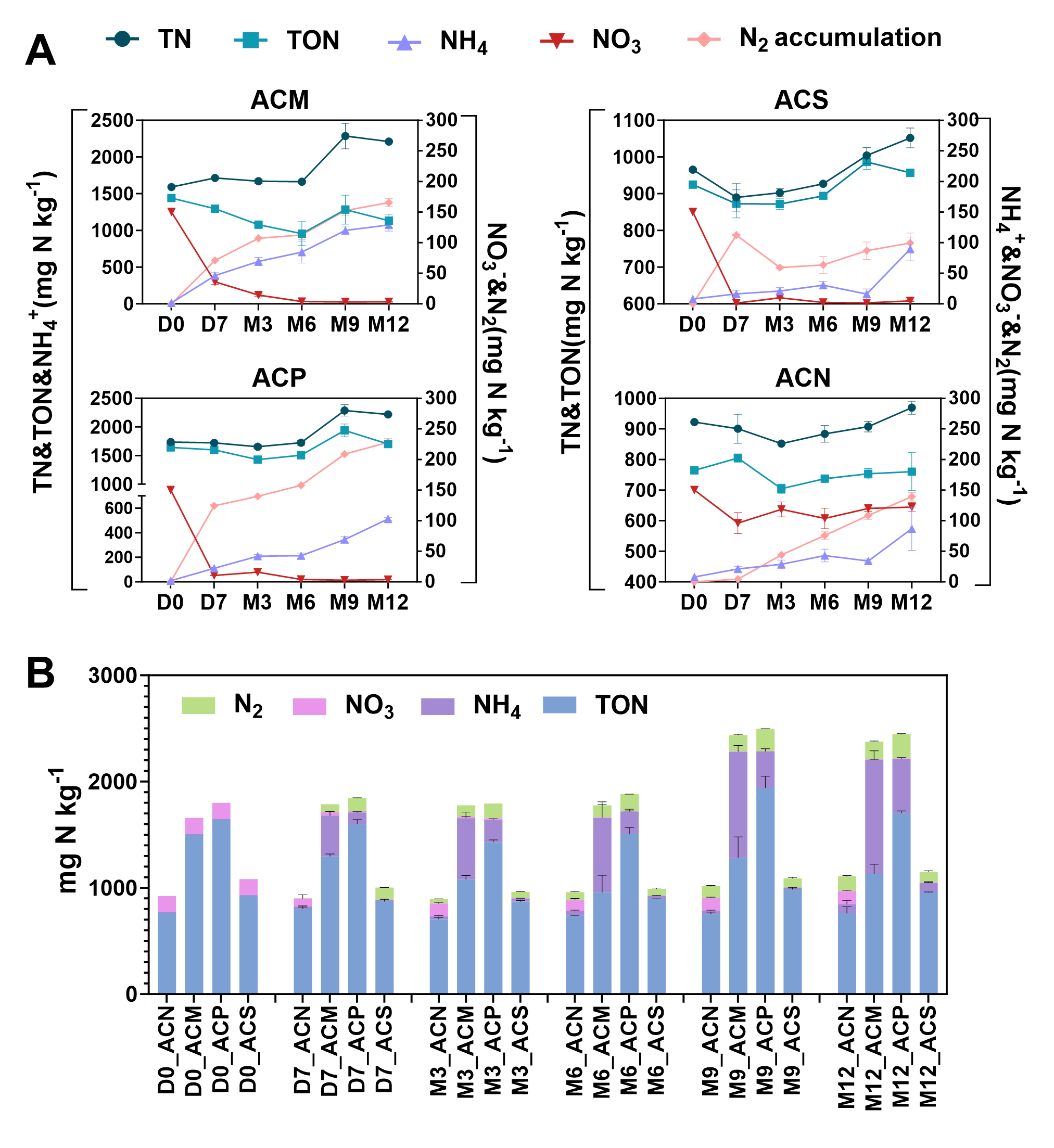


**Supplementary Figure S5** Changes of nitrogen-related physicochemical parameters and estimation of nitrogen balance in each group during incubation period


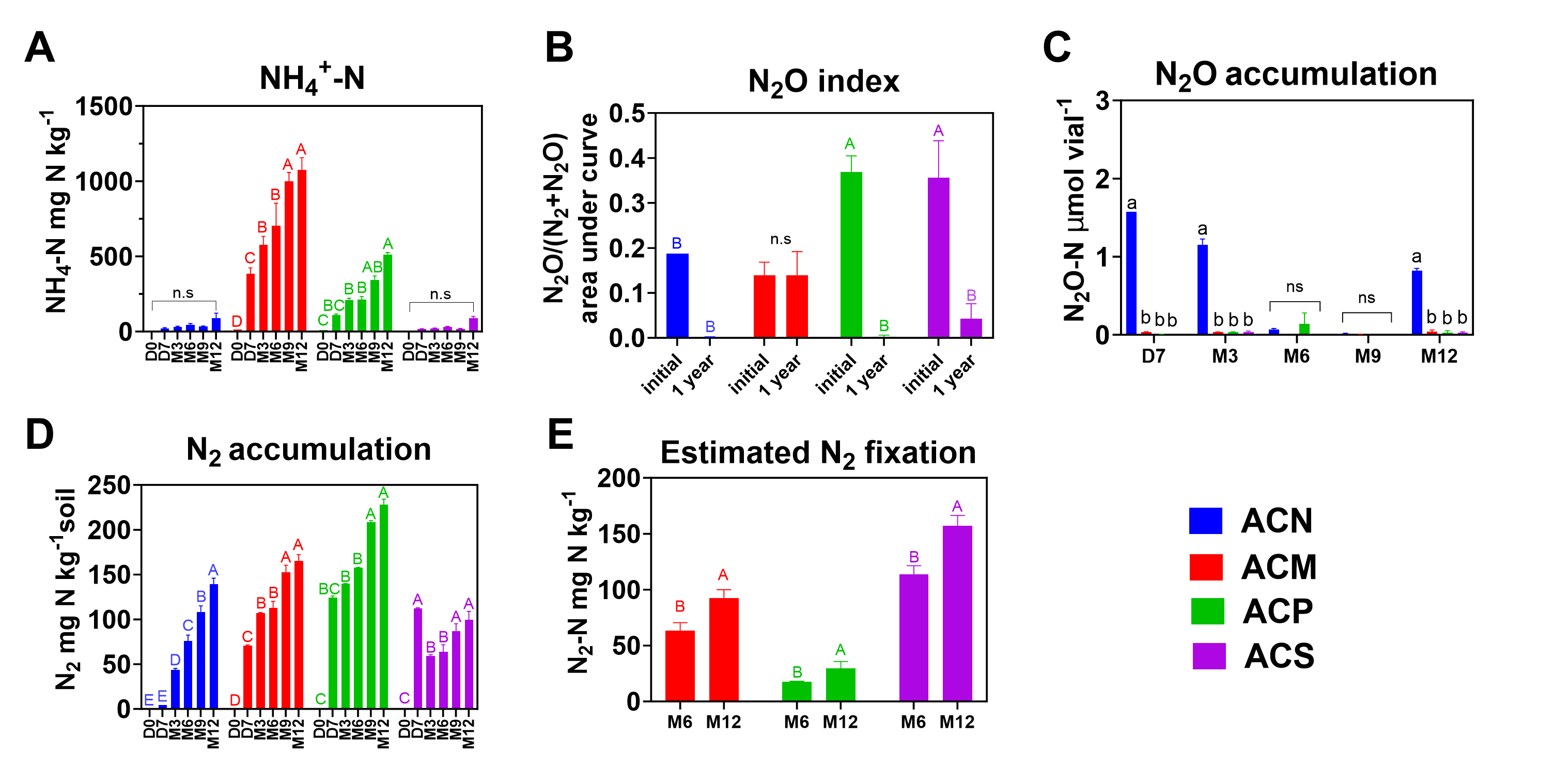


**Supplementary Figure S6** Changes in NH₄⁺-N, N₂ accumulation, N₂O index, N₂O accumulation, and estimated N₂ fixation in experimental groups over a 1-year anaerobic incubation.

(A) NH₄⁺-N concentrations, (B) N₂O index based on N₂O/(N₂ + N₂O) area under the curve, (C) N₂O accumulation (D) N₂ accumulation, and (E) estimated N₂ fixation are shown for four treatment groups: ACN (control), ACM (anaerobically fermented pig manure digestate), ACP (aerobically fermented pig manure compost), and ACS (straw powder). Samples were collected at multiple time points (0 days, 7 days, 3 months, 6 months, 9 months, and 12 months) as part of a parallel, destructive sampling strategy. Due to the destructive nature of sampling and extended time intervals, repeated measures were not applied in Two-way ANOVA. Capital letters indicate significant differences within groups across different time points (using standard Two-way ANOVA followed by Tukey’ s HSD post hoc test). Error bars represent the standard error of the mean (n=3). A global summary of inter-time point and inter-group significance is provided in Supplementary Tables S8.

The ACN group showed noticeable N₂O accumulation, particularly at the D7 and M12 time points, while the ACM, ACP, and ACS groups maintained undetectable or minimal N₂O levels, suggesting enhanced denitrification activity in these treatments that likely reduced N₂O to N₂.


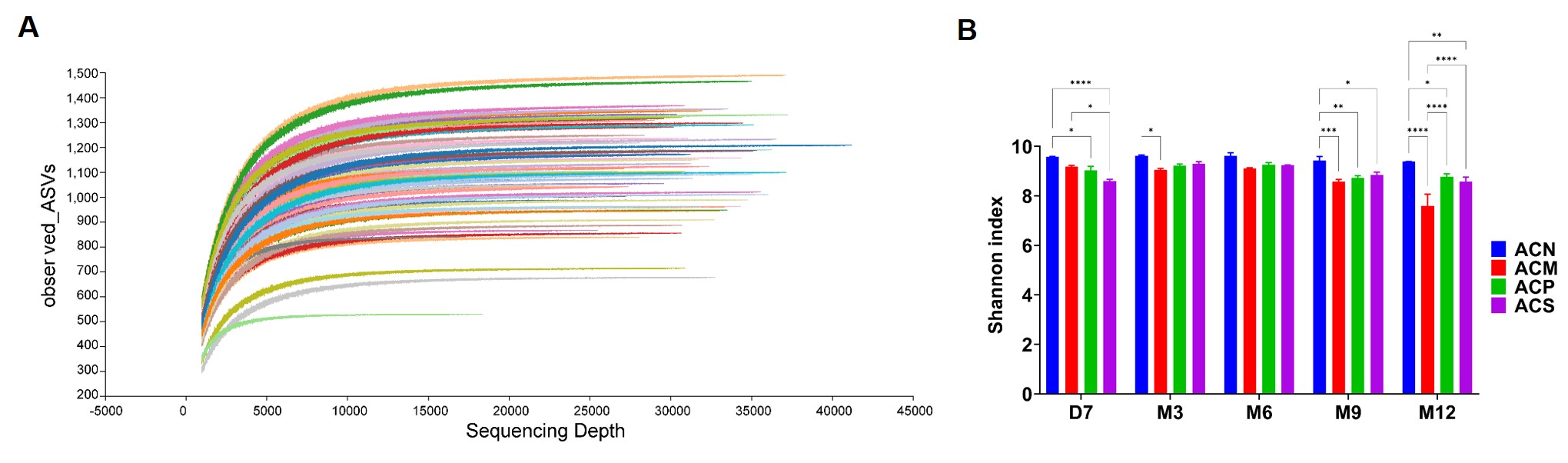


**Supplementary Figure S7** Alpha rarefaction curves and changes in Shannon index for microbial communities after organic fertilizer addition.

**(A)** Alpha rarefaction curves for high-throughput sequenced sequences in the 16S v3-v4 region of all samples. The rarefaction curves eventually flattened, indicating that the sequencing depth was sufficient to meet the requirements of subsequent data analysis. **(B)** Changes in Shannon index for each group after the addition of organic fertilizer. The Shannon index of the soil microbial communities decreased after the addition of organic amendments, suggesting that while the organic amendments enriched some functional microbial communities, the species diversity of the overall community decreased.


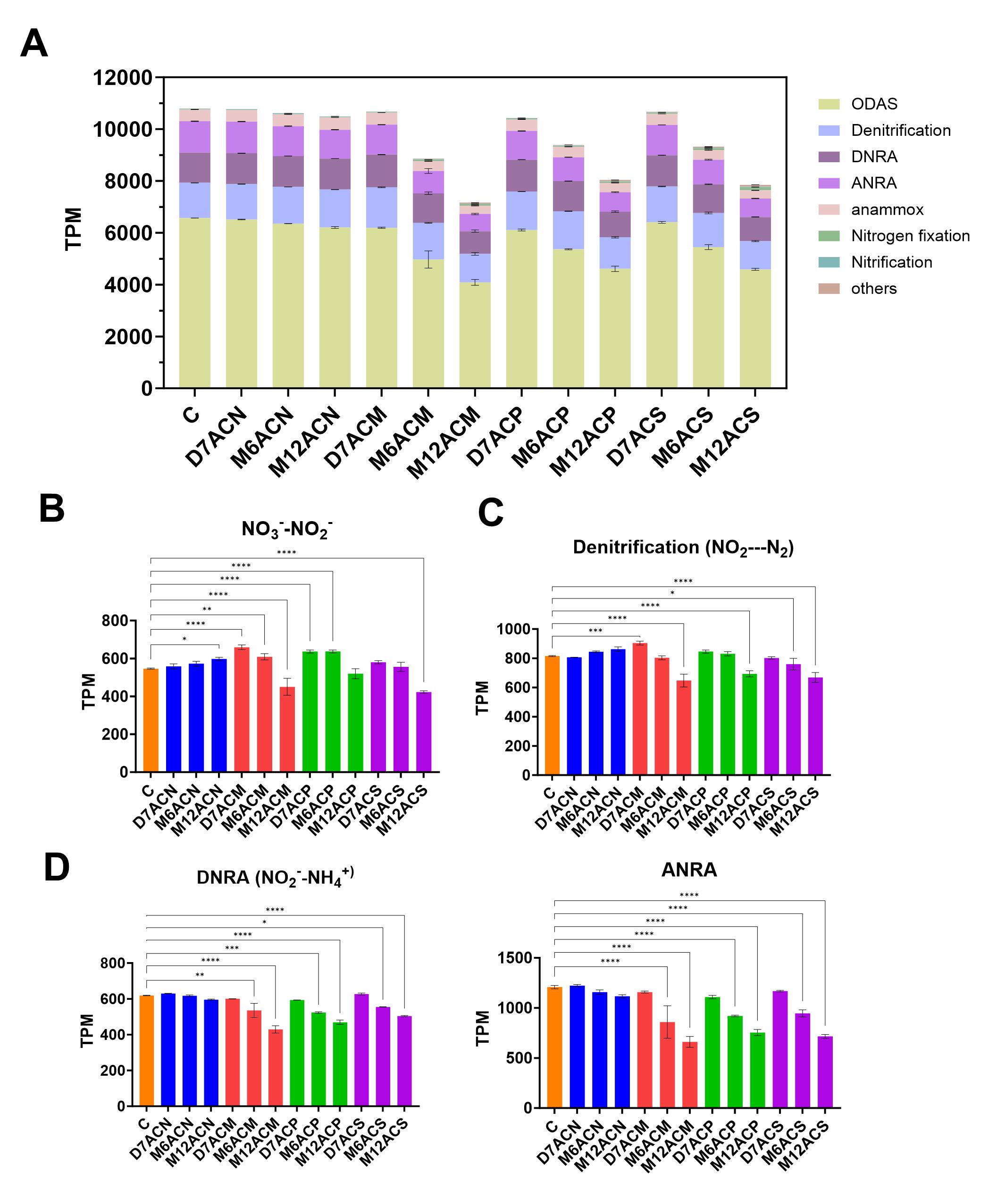


**Supplementary Figure S8** Abundance dynamics of nitrogen cycling pathways during anaerobic incubation.

(A) Stacked bar plot illustrating the total TPM (transcripts per million) of nitrogen cycling pathways, including Organic Degradation and Synthesis (ODAS), Denitrification, Dissimilatory Nitrate Reduction (DNRA), Assimilatory Nitrate Reduction (ANRA), Anammox, Nitrogen Fixation, Nitrification, and Others, across all treatments and time points. (B) Bar plot showing the summed TPM of functional genes involved in nitrate reduction to nitrite, a process shared by DNRA and denitrification pathways. (C) Summed TPM of genes involved in nitrite reduction to nitrogen gas in the denitrification pathway. (D) Summed TPM of genes responsible for nitrite reduction to ammonium in the DNRA pathway. (E) Summed TPM of all genes in the ANRA pathway. Statistical significance was assessed using one-way ANOVA followed by Dunnett's post hoc test, comparing amended groups (ACM and ACP) to the control group (ACN). Error bars represent standard deviations. Asterisks indicate statistical significance (*P < 0.05, **P < 0.01, ***P < 0.001, ****P < 0.0001).





**Supplementary Figure S9** Relative abundances for 68 nitrogen cycling genes during cultivation. Differences in the values were calculated via two-way ANOVA. Different letters indicate significant difference (p < 0.05) among timepoint of each group (n=3)


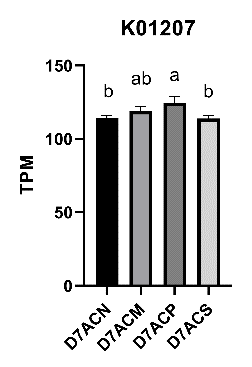

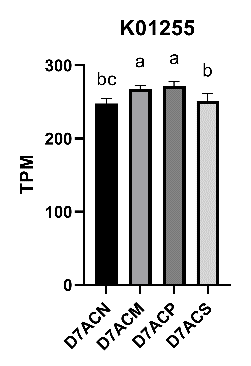


**Supplementary Figure S10** Abundance of two N-acquiring enzyme encoding genes at the D7 time point.

The abundances of the nitrogen-acquiring enzyme-encoding genes *K01255* (leucine aminopeptidase, LAP) and *K01207* (β-1,4-n-acetylglucosaminidase, NAG) during the early incubation stage. Differences in the values were calculated via two-way ANOVA. Different letters indicate significant difference (P < 0.05) of each group (n=3)


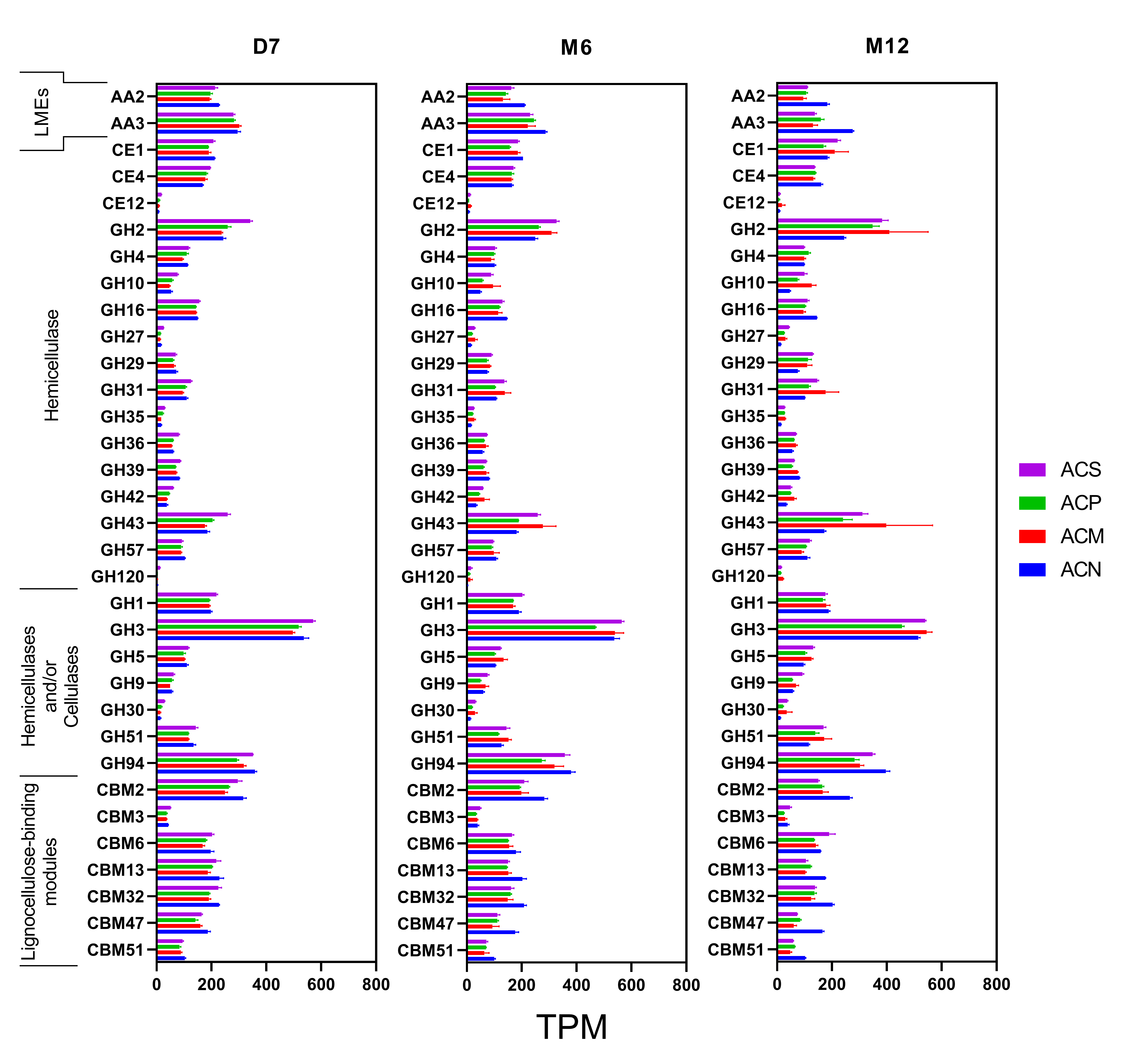


**Supplementary Figure S11** Cellulose, lignin and hemicellulose decomposition related CAZymes that differed among groups.

At D7 the ACS group was enriched for 14 cellulose and/or hemicellulose decomposition associated CAZymes (CE4, GH2, GH10, GH27, GH31, GH35, GH36, GH42, GH43, GH1, GH3, GH30, GH51, CBM3). At 6M the ACS group was enriched for 8 cellulose and/or hemicellulose catabolism-related CAZymes (GH2, GH10, GH31, GH42, GH43, GH3, GH5, GH51). At 12M the ACS group was enriched for 9 cellulose and/or hemicellulose catabolism-related CAZymes (CE1, GH2, GH10, GH29, GH31, GH43, GH5, GH9, GH51)


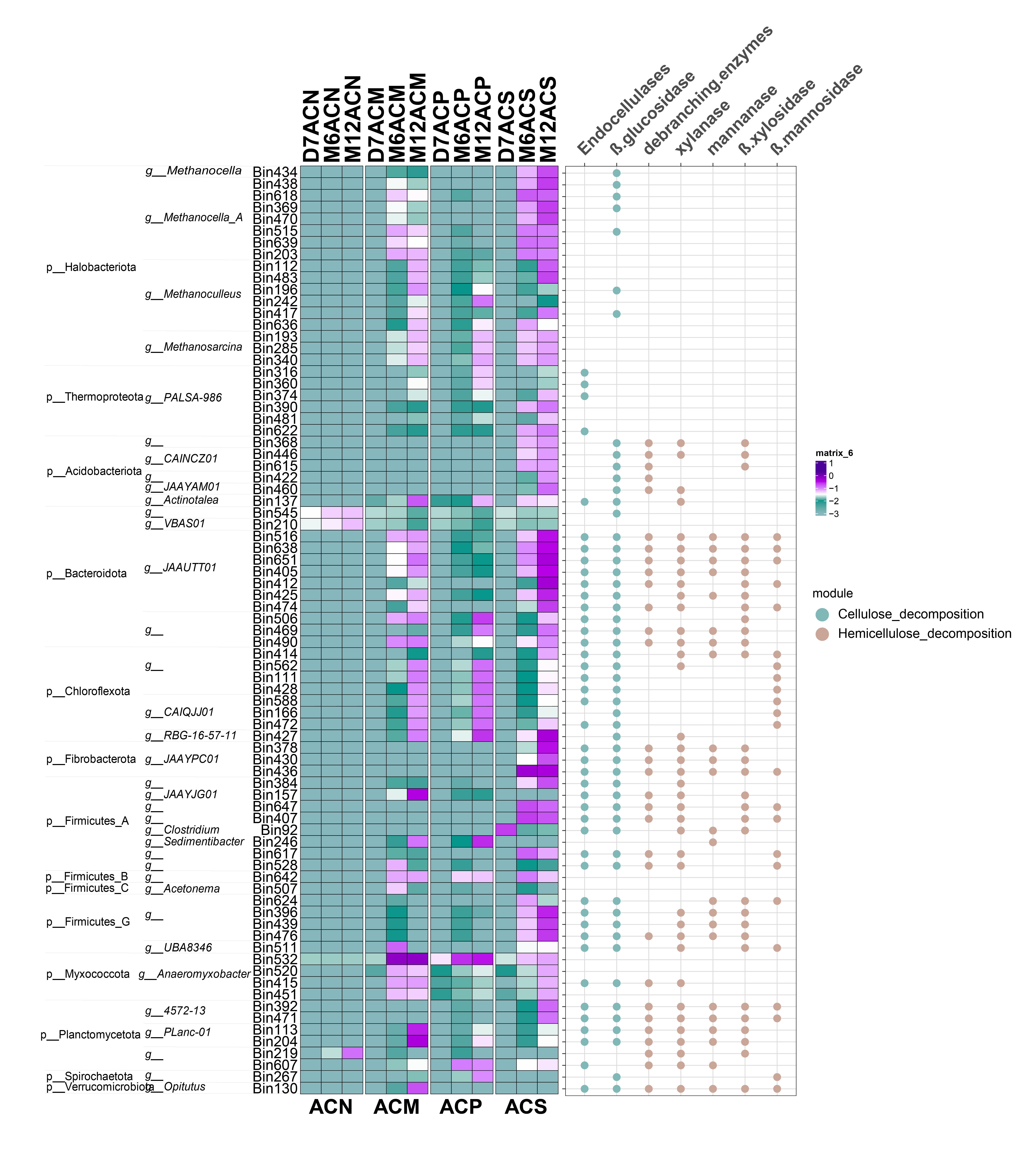


**Supplementary Figure S12** Annotation information for genes encoding cellulose and hemicellulose degrading enzymes of 79 nitrogen-fixing MAGs


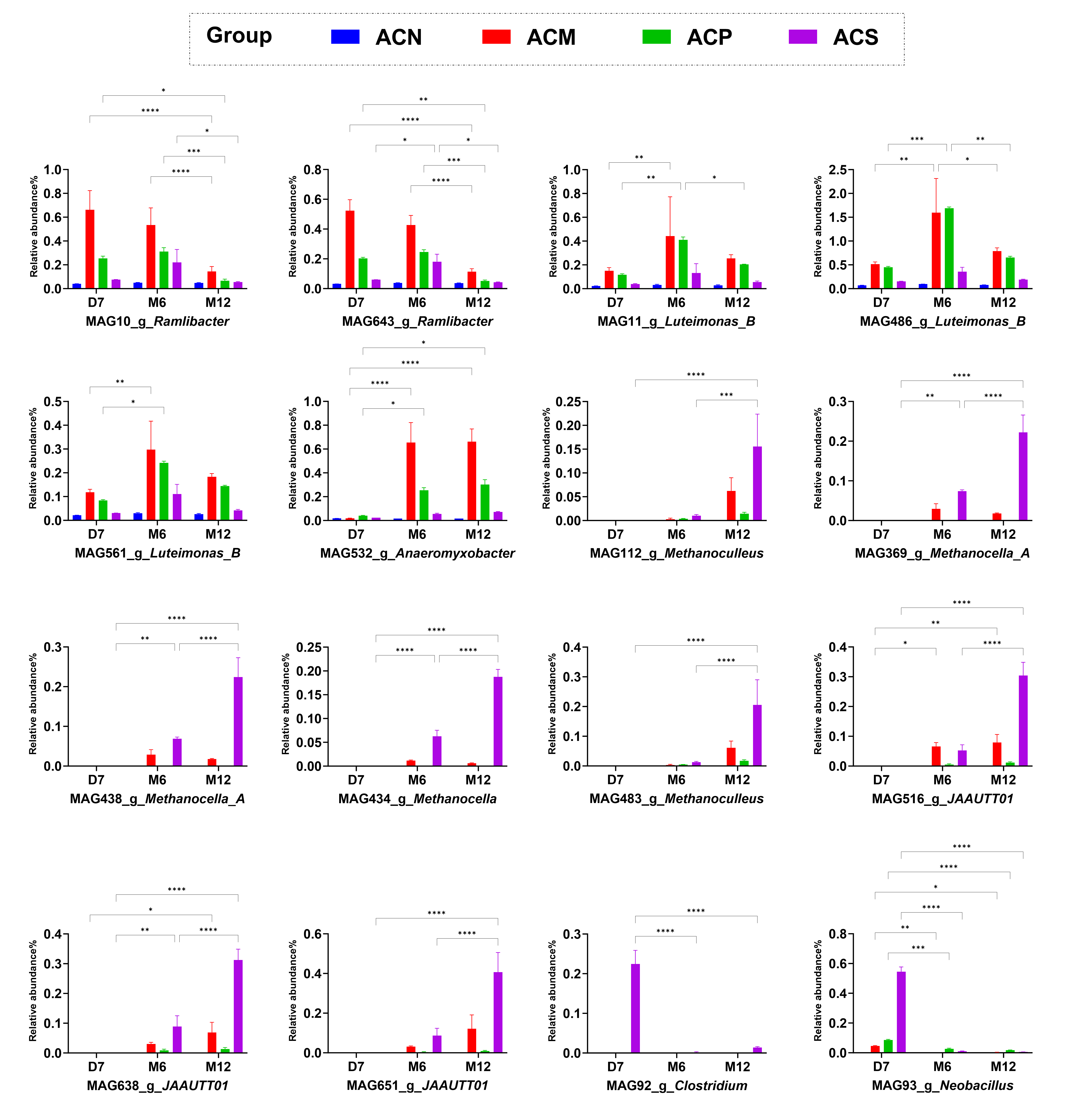


**Supplementary Figure S13** Relative abundance of key MAGs selected by random forest analysis, showing temporal changes in abundance across experimental groups. Error bars indicate the standard error of the means (n = 3). Differences in the values were calculated based on analysis of variance (ANOVA) and Tukey’s HSD post hoc test. Asterisks indicate statistical significance (*P < 0.05, **P < 0.01, ***P < 0.001, ****P < 0.0001).

**Supporting information reference**

Huang T, Yang H, Huang C, Ju X (2017) Effect of fertilizer N rates and straw management on yield-scaled nitrous oxide emissions in a maize-wheat double cropping system. Field Crops Res 204:1-11. https://10.1016/j.fcr.2017.01.004

Song X, Ju X, Topp CFE, Rees RM (2019) Oxygen Regulates Nitrous Oxide Production Directly in Agricultural Soils. Environ Sci Technol 53:12539-12547. https://10.1021/acs.est.9b03089

Molstad, L., Dörsch, P., & Bakken, L. R. (2007). Robotized incubation system for monitoring gases (O2, NO, N2O N2) in denitrifying cultures. Journal of microbiological methods, 71(3), 202-211.
